# DPP‐DTT Nanowire Phototransistors for Optoelectronic Synapses in EMG and ECG Signal Classification

**DOI:** 10.1002/smll.202506440

**Published:** 2025-08-08

**Authors:** Wangmyung Choi, Jin Seok Yoon, Won Woo Lee, Gun Ho Hong, Hyeonjung Kim, Seyong Oh, Young Tea Chun, Hocheon Yoo

**Affiliations:** ^1^ Department of Electronic Engineering Hanyang University 222 Wangsimni‐ro Seoul 04763 Republic of Korea; ^2^ Division of Electronics and Electrical Information Engineering Korea Maritime and Ocean University 727 Taejong‐ro Busan 49112 Republic of Korea; ^3^ Department of Artificial Intelligence Semiconductor Engineering Hanyang University 222 Wangsimni ‐ ro Seoul 04763 Republic of Korea; ^4^ Division of Electrical Engineering Hanyang University ERICA 55 Hanyangdaehak‐ro Ansan 15588 Republic of Korea

**Keywords:** DPP‐DTT nanowire, electrocardiogram, electromyography, neuromorphic device, photo‐gating effect, phototransistor, physiological signal classification

## Abstract

A neuromorphic phototransistor based on nanowire‐patterned diketopyrrolo‐pyrrole‐dithienylthieno[3,2‐b]thiophene (DPP‐DTT) is reported. The nanowires, well‐aligned with a width of 460 nm, spacing of 8–11 µm, and height of ≈80 nm, are fabricated using the stamping method of soft lithography and exhibit optically stimulated synaptic behavior. Under blue illumination (455 nm, 0.55 mW cm−2), a photogating effect arises at the DPP‐DTT/SiO2 interface, leading to threshold voltage shifts up to 6.4 V as a result of electron trapping at the interface. Negative gate pulses (−7 V) facilitate recombination of the trapped electrons, inducing detrapping and consequently leading to a decrease in the threshold voltage. These two behaviors effectively emulate the processes of potentiation and depression. Efficient trap–detrapping dynamics are facilitated by the unique geometry of the nanowire. Synaptic plasticity is modulated by adjusting stimulus intensity (light pulse: 0.26–1.42 mW cm^−2^, gate pulse: −6–−9 V), duration (0.3–2.1 s), frequency (0.47–3.33 Hz), and repetition (1–40 cycles), supporting transitions from short‐ to long‐term behavior. The device is evaluated through artificial intelligence classification tasks, including image recognition and time‐dependent physiological analysis. It achieves the classification accuracies of 97.4% for MNIST, 93.4% for electromyography (7 classes), 89.0% for electrocardiography (5 classes), and 83.8% for CIFAR‐10.

## Graphical abstract

A DPP‐DTT NW‐based PT emulates synaptic responses to both optical and electrical inputs, enabling a hardware platform for neuromorphic computing. The proposed device achieved 88.3% and 71.6% classification accuracy on EMG (7 classes) and ECG (5 classes), respectively, highlighting its potential for real‐time biosignal processing in wearable and edge neuromorphic applications.

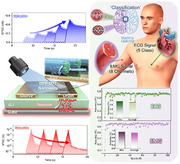

## 1 Introduction

Optically modulated synaptic devices represent a promising route for neuromorphic systems that require low‐power operation, precise spatial control, and parallel optical stimulation.^[^ ^1^, ^2^ ^]^ Light stimulation provides a non‐invasive means of adjusting synaptic weights (SWs), with tunability across wavelength, intensity, and duration, features that are difficult to achieve with purely electrical schemes.^[^ ^3^, ^4^, ^5^, ^6^, ^7^ ^]^ These advantages make light‐based synapses particularly appealing for multi‐channel neuromorphic circuits.

Organic semiconducting polymers offer unique advantages for neuromorphic device fabrication, including mechanical flexibility, energy band tuning, and compatibility with low‐cost, solution‐based processing.^[^ ^8^, ^9^, ^10^, ^11^, ^12^, ^13^ ^]^ These characteristics enable large‐area manufacturing without the need for high‐temperature or vacuum‐based techniques.^[^ ^14^ ^]^ Among various patterning strategies, soft lithographic approaches such as stamping allow for the direct formation of micro‐ or nanoscale features with well‐defined geometries, eliminating the need for subtractive etching or photolithography.^[^ ^15^, ^16^, ^17^ ^]^ This capability is particularly valuable in constructing anisotropic channel structures, such as nanowire arrays.^[^ ^18^, ^19^, ^20^ ^]^

Nanowire (NW) architecture provides additional advantages for emulating neural connectivity.^[^ ^21^, ^22^, ^23^ ^]^ By controlling the number, spacing, and orientation of the polymer wires, it becomes possible to define discrete, spatially organized conduction paths analogous to synaptic connections between neurons. Diketopyrrolo‐pyrrole‐dithienylthieno[3,2‐b]thiophene (DPP‐DTT), a donor–acceptor conjugated polymer, exhibits strong π–π stacking interactions and a rigid, planar backbone, which facilitate efficient charge transport and stable molecular alignment in NW configurations.^[^ ^24^, ^25^, ^26^, ^27^, ^28^ ^]^ These structural characteristics make DPP‐DTT particularly well‐suited for forming highly crystalline and aligned NW architectures through solution‐based patterning techniques. This structured arrangement not only facilitates reproducible device characteristics but also enhances charge transport along the wire axis.^[^ ^29^, ^30^, ^31^, ^32^, ^33^, ^34^, ^35^ ^]^ Furthermore, the interface between NW and underlying dielectric hosts trap sites that enable photogating‐driven modulation of channel conductance.^[^ ^36^, ^37^, ^38^ ^]^ Under light exposure, photoexcited carriers are trapped at these interfaces, shifting the threshold voltage and increasing channel current, an analog to synaptic potentiation.^[^ ^39^ ^]^ Application of negative gate bias induces detrapping and recombination, resulting in depression behavior.^[^ ^40^, ^41^, ^42^ ^]^ These dynamics allow the device to emulate bidirectional synaptic plasticity in response to optical and electrical stimuli.

To exploit this neuromorphic potential, we present a solution‐processed phototransistor (PT) based on DPP‐DTT NWs that functions as a light‐responsive artificial synapse. The DPP‐DTT NWs structure, fabricated via a stamping‐assisted technique, enables high crystallinity and clean interfaces across well‐defined conduction paths.^[^ ^32^ ^]^ The device exhibits robust photogating behavior, with synaptic plasticity modulated by optical intensity, duration, frequency, and gate bias conditions. We demonstrate bidirectional synaptic behavior, including potentiation and depression, by means of trap–detrapping dynamics at the DPP‐DTT/SiO_2_ interface. DPP‐DTT based PT devices adopted a heterojunction structure or polymer electret to facilitate the separation and trapping of photoexcited carriers. Wu, et al. utilized poly(2‐(3′,3′‐dimethyl6‐nitrospiro[chromene‐2,2′‐indolin]‐1′‐yl) ethyl methacrylate) as a ultraviolet (UV) light‐assisted charge trapping layer by heterojunction with DPP‐DTT.^[^ ^43^ ^]^ Zhao et al. developed a DPP‐DTT‐based PT using a polymer electret composed of phenyl‐C61‐butyric acid methyl ester and polyacrylonitrile, which served as a photocarrier trapping layer and enabled UV‐stimulated optical synaptic behavior.^[^ ^44^ ^]^ However, these previous studies depended on UV‐responsive materials blended with DPP‐DTT, which caused structural complexity and deteriorated device performance. The proposed PT is solely based on pristine DPP‐DTT NWs, enabling stable photocarrier trapping under visible light illumination and offering advantages in both fabrication simplicity and operational reliability. The proposed DPP‐DTT NW‐based PT with a neural network obtained a training maximum accuracy of 97.4% for handwritten digit classification (Modified National Institute of Standards and Technology, MNIST). We also implement classification of physiological signals, achieving training accuracies of 93.4% for electromyography (EMG), 89.0% for electrocardiography (ECG), and 83.8% for object recognition (Canadian Institute for Advanced Research‐10, CIFAR‐10).

## 2 Results and Discussion

**Figure 1:**
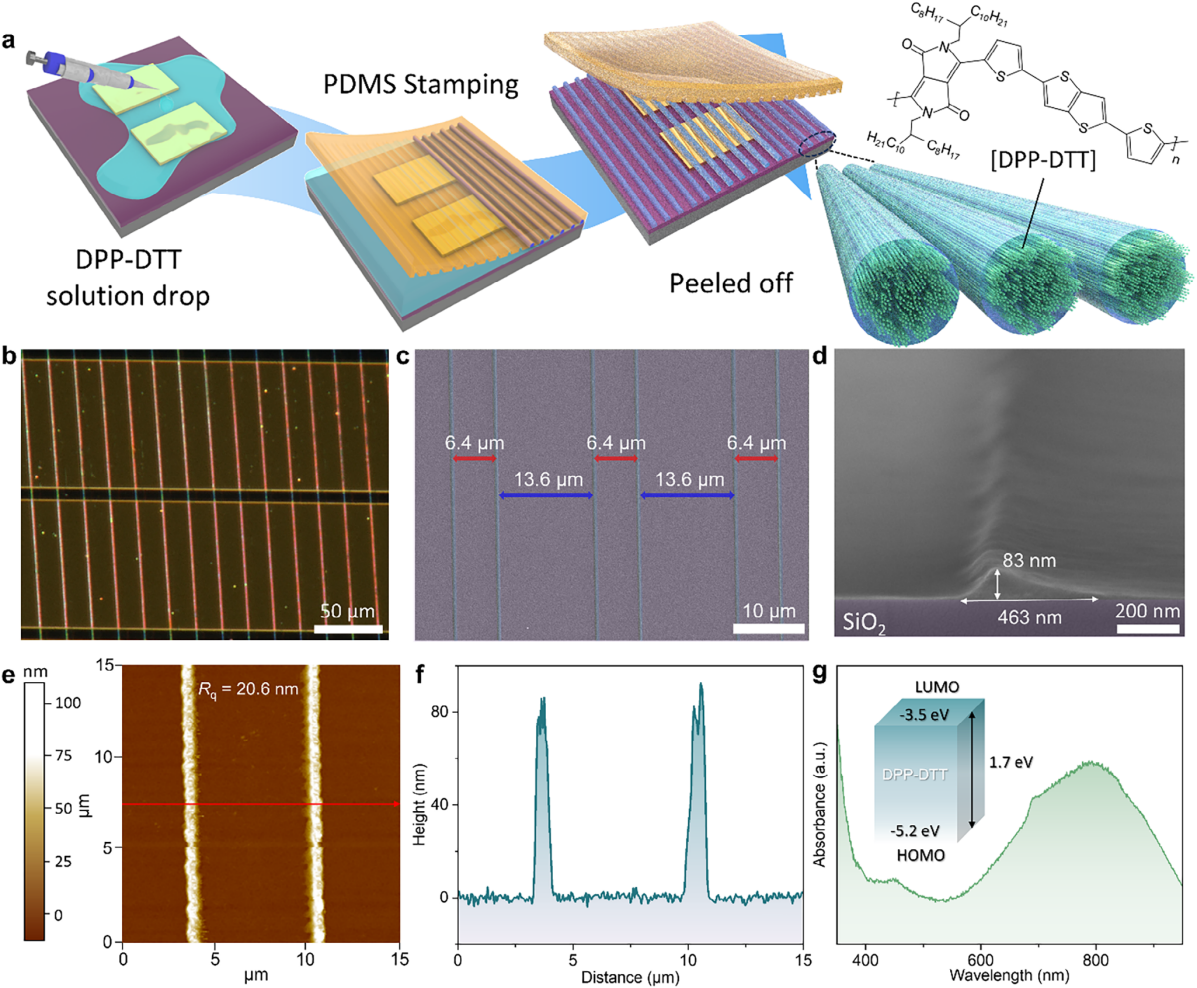
a) Schematic illustration of the PT based on DPP‐DTT NWs for fabrication process and formation of DPP‐DTT NWs via soft lithography. Morphology information of DPP‐DTT NWs. b) OM image of the DPP‐DTT NWs‐based PT with a distance between the electrodes of 10 µm (scale bar is 50 µm). c) SEM surface image of the DPP‐DTT NWs (scale bar is 10 µm). The NW spacing in the groove and ridge regions is 6.4 and 13.6 µm, respectively. d) SEM cross section image of the single DPP‐DTT NW (scale bar is 200 nm). e) AFM topography of the two DPP‐DTT NW at the 15 µm × 15 µm scale (the root‐mean‐square roughness is 20.6 nm). f) Height profiles of the two DPP‐DTT NWs extracted from the AFM topography (the spacing and height of the NWs were 6.8 µm and 80 nm, respectively). g) Absorption spectra of the DPP‐DTT NWs (inset: energy band diagram information of the DPP‐DTT NWs).

A DPP‐DTT NWs‐based PT was fabricated via a solution‐based soft lithography method using patterned polydimethylsiloxane (PDMS) stamps (**Figure** **1**a). NWs pattern‐formation principle is schematically illustrated in Figure S1 (Supporting Information). A DPP‐DTT solution was cast onto the SiO_2_ substrate, and a PDMS template with microscale grooves was then gently placed on top (Figure S1a, Supporting Information). As the solvent gradually evaporated at room temperature, the solution became confined within the grooves, and the curved liquid surface was pinned to the sidewalls of the PDMS structure.^[^ ^45^ ^]^ This led to the formation of capillary bridges at the interface between the groove sidewalls and the substrate, which served as nucleation sites for NW growth (Figure S1b, Supporting Information). Over time, DPP‐DTT NWs formed along these triangular menisci via capillary‐guided self‐assembly (Figure S1c, Supporting Information). After solvent evaporation, the NWs remained aligned within the capillary bridge regions. As a result, the inter‐wire distance in the groove region (D_1_) was smaller than that in the ridge region (D_2_) (Figure S1d, Supporting Information). Figure 1b shows a top‐view optical microscopy (OM) image of the resulting device. The DPP‐DTT NWs were uniformly aligned across the Au electrodes, forming a continuous array without visible cracks or polymer residue. To examine the morphology of the DPP‐DTT NWs, scanning electron microscopy (SEM) was conducted. The DPP‐DTT NWs exhibited a uniform spacing of 6.4 µm in the groove regions, while those formed in the ridge regions showed a consistent spacing of 13.6 µm (Figure 1c). Their height and width were measured to be 83 and 463 nm, respectively (Figure 1d). In terms of the morphology and polymer residue of the DPP‐DTT NWs, energy‐dispersive spectroscopy (EDS) line scanning was performed over a 32 µm range on both a bare SiO_2_ substrate and a SiO_2_ substrate with DPP‐DTT NWs, as shown in Figure S2a,b (Supporting Information). The bare SiO_2_ substrate exhibited a carbon intensity of ≈10 cps, which can be attributed to baseline (Figure S2c, Supporting Information). When DPP‐DTT NWs were deposited on the SiO_2_ surface (Figure S2d, Supporting Information), four sharp carbon intensity peaks were observed at the same positions as the NWs shown in Figure S2b (Supporting Information). The intensity between each peak remained at the baseline as that of the bare substrate, indicating that the NWs were independently isolated without polymer residues. This result was further supported by cross‐sectional SEM analysis. As shown in Figure S3a (Supporting Information), two isolated NWs formed within the groove region are clearly visible. A cross‐sectional image across a single NW spanning both the groove (Region 1) and ridge (Region 2) reveals a smooth interface without any residue, confirming the separation of the NWs (Figure S3b,c, Supporting Information). To validate the pattern uniformity of the DPP‐DTT NWs and confirm the absence of polymer residues, atomic force microscopy (AFM) analysis was performed. As shown in Figure 1e, the root‐mean‐square roughness (*R*_q_), measured to be 20.6 nm across both the groove and ridge regions in the AFM topography, along with the similar height observed between these regions (brown areas), suggests negligible residual polymer between the NWs. Figure 1f further confirms the morphological consistency, with NW spacing of 6.8 µm and a height near 80 nm in the groove region, in agreement with the SEM measurements. The optical and electronic properties of the DPP‐DTT NWs were characterized through UV–vis absorption spectroscopy and energy level analysis. As shown in Figure 1g, the NWs exhibit broadband absorption from 400 nm to 900 nm, consistent with their role as a p‐type organic semiconductor. The material features a HOMO level of −5.2 eV, LUMO level of −3.5 eV, and an optical bandgap of 1.7 eV.^[^ ^43^, ^46^ ^]^ This solution‐based pattering technique demonstrates the reproducible nanoscale manufacture of organic semiconductors without high‐temperature and vacuum processes.

**Figure 2:**
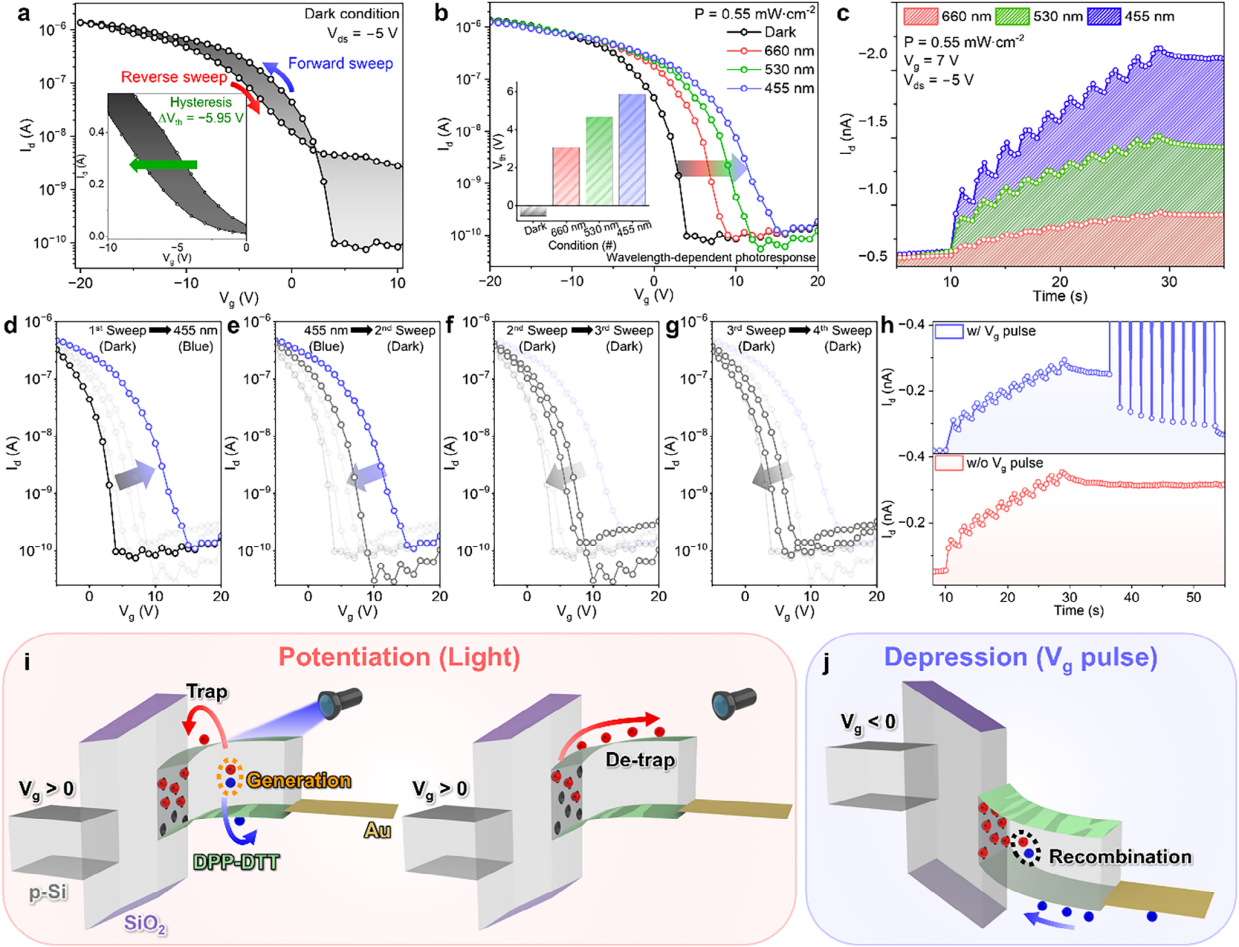
a) Hysteresis caused in the transfer curve of the DPP‐DTT NWs‐based PT as a function of the gate voltage sweep direction (inset: linear scale). *V*_ds_ was fixed at −5 V. b) Transfer curve of the DPP‐DTT NWs‐based PT under various measurement conditions with a fixed *V*_ds_ of −5 V (inset: histogram of threshold voltage under different measurement conditions). c) Synaptic potentiation of the DPP‐DTT NWs‐based PT at irradiated ten light pulses of 455, 530, and 660 nm. Current‐time characteristics were measured at −5 V *V*_ds_ and 7 V *V*_g_, with light pulses of 0.55 mW cm^−2^ applied for 1 s. d) and g) Step‐by‐step transfer characteristics of the DPP‐DTT NWs‐based PT under various measurement conditions with a fixed *V*_ds_ of −5 V. h) Synaptic depression of the DPP‐DTT NWs‐based PT under constant −5 V *V*_ds_ and 7 V *V*_g_, comparing conditions with and without a negative gate pulse. Light pulses (0.55 mW cm^−2^, 1 s) and gate pulses (−1 V, 0.3 s) were applied. Mechanism of synaptic (i) potentiation and (j) synaptic depression induced individual 455 nm light and gate voltage pulses.

The electrical properties of the DPP‐DTT NWs‐based PT were systematically analyzed to elucidate the origin of its synaptic characteristics. During the electrical characterization, the drain‐source voltage (*V*_ds_) was fixed at −5 V. **Figure** **2**a presents the transfer characteristics under dark conditions, showing a negative shift of approximately ∆*V*_th_ = −5.95 V and gate voltage‐dependent hysteresis. This behavior arises from carrier trapping by OH^−^ functional groups at the DPP‐DTT NWs/SiO_2_ interface. Upon illumination with 660 (red), 530 (green), and 455 nm (blue) light at a fixed intensity (0.55 mW cm^−2^), the transfer curves shifted in the positive direction, with larger shifts at shorter wavelengths (Figure 2b). The threshold voltage (*V*_th_) increased from −0.52 V (dark) to 5.87 V (blue light), as shown in Figure S4 (Supporting Information), indicating a photogating effect driven by photoexcited‐electron trapping at the interface. Interestingly, despite stronger UV–vis absorption in the red region (Figure 1g), the device exhibited greater photo‐responsivity to blue light. This inversion is attributed to the use of 1,2‐dichlorobenzene (DCB), which partially removes OH^−^ groups from the SiO_2_ surface and renders it hydrophobic,^[^ ^47^ ^]^ enhancing blue light response.^[^ ^48^ ^]^ To verify that DCB partially removes OH^−^ functional groups from the SiO_2_ interface, we conducted contact angle measurements and X‐ray photoelectron spectroscopy (XPS) analysis (Figure S5, Supporting Information). As a result, the contact angle increased from 60.01° to 99.23° after surface treatment, and the proportion of OH^−^ bonding, as quantified by XPS, decreased from 71.3% to 39.6%. This indicates that the DCB treatment clearly decreases the density of OH^−^ functional groups on the SiO_2_ surface, but does not completely eliminate them, allowing for potential carrier trapping. Figure 2c shows the time‐resolved photoresponse under constant illumination for red, green, and blue wavelengths. In all cases, photoexcited‐electrons are trapped, producing synaptic‐like responses, with enhanced behavior under blue light due to more efficient carrier trapping and stronger photogating effects. To investigate the detrapping dynamics, transfer curves were sequentially measured under various conditions (Figure 2d–g). After illumination, the curve shifts right (Figure 2d), but successive sweeps in the dark restore the *V*_th_ to its original state (Figure 2e–g). This recovery is enabled by the application of negative gate bias at the end of the forward sweep, which facilitates recombination between trapped electrons and injected holes at the interface. This dynamic recovery mechanism is critical for implementing inhibitory synaptic functions. To investigate the role of geometry in enhancing the photogating effect, we focused on the NW‐based DPP‐DTT PT, which exhibited a significantly stronger photoresponse. To provide a meaningful comparison, a thin‐film DPP‐DTT PT was fabricated under identical conditions (Figure S6, Supporting Information). A systematic comparison of their electrical characteristics revealed that the NW and thin‐film devices exhibited hysteresis values of 5.95 and 5.15 V, subthreshold swings of 1.54 and 1.41 V dec^−1^, and interface trap densities (*D*_it_) of 5.38 × 10^12^ and 4.89 × 10^12^ cm^−2^ eV^−1^, respectively. These results indicate that the NW structure contains more localized interfacial traps. The high trap density in the NW structure can enhance the photo‐gating effect. Upon light illumination, photo‐generated carriers interact with the densely distributed interfacial traps, resulting in stronger local electric fields and a more pronounced *V*_th_ shift. As shown in Figure S7a,b (Supporting Information), under 455 nm light at an intensity of 0.55 mW cm^−2^, the NW device exhibits a light‐induced *V*_th_ shift of 6.39 V, compared to 2.25 V in the thin‐film device. This significant difference indicates that the higher trap density concentrated in the smaller channel area of the NW leads to a stronger photo‐gating effect due to enhanced local field modulation. To further support this conclusion, we also calculated the photoresponsivity using the following expression:

(1) $$Photoresponsivity=\frac{\left(J_{\mathrm{photo}}-J_{\mathrm{dark}}\right)}{P_{\mathrm{light}}}$$

where *J*_photo_ and *J*_dark_ are the drain current density under illumination and dark, respectively, and *P*_light_ is the incident optical power. The photoresponsivity was calculated using the *J*_photo_ and *J*_dark_ extracted at *V*_g_ = 4 V and *V*_g_ = 3 V for the NW and thin‐film devices, respectively, corresponding to their maximum photoresponses. The calculated photoresponsivity values were 2.54 × 10^−2^ A W^−1^ for the NW device and 6.08 × 10^−6^ A W^−1^ for the thin‐film device, clearly demonstrating that the NW exhibits a significantly enhanced photoresponse. Since photoresponsivity inherently reflects the effective channel area, this result further supports that the NW structure benefits from a higher local trap density and stronger photo‐gating modulation per unit area. We summarized the trap‐dependent parameters of the NW and thin‐film devices in Table S1 (Supporting Information). Figure 2h compares synaptic responses with and without a negative gate pulse. Without the pulse, the photocurrent remains unchanged after illumination. Conversely, applying a negative gate pulse after the light pulse causes a gradual decrease in drain current, confirming synaptic depression functionality. These results demonstrate that the DPP‐DTT NWs‐based PT can implement both potentiation and depression via coordinated light and gate pulse control. For potentiation (Figure 2i; Figure S8, Supporting Information), blue light generates electron–hole pairs in the channel. Under positive gate bias (*V*_g_ > 0), the photoexcited electrons are trapped at the DPP‐DTT NW/SiO_2_ interface due to the downward band bending, while the holes are transported to the Au electrode (Figure S8a, Supporting Information). The trapped photoexcited electrons induce a local electric field that acts as an additional negative gate bias, enhancing the photogating effect and increasing the drain current. After the light is turned off, electrons trapped at the interface exhibit partial detrapping, resulting in a slight decrease in the drain current (Figure S8b, Supporting Information). When the device is subsequently exposed to light, additional photoexcited electrons accumulate at the interface, leading to an increased drain current (Figure S8c, Supporting Information). This process enables SW modulation through repetitive light exposure. To further confirm the gate‐dependent tunability of synaptic potentiation, the photoresponse and memory characteristics of the DPP‐DTT NW‐based PT were systematically investigated by analyzing current‐time characteristics under *V*_g_ = 0 V and *V*_g_ = 7 V (Figure S9a, Supporting Information). Ten light pulses of 0.55 mW cm^−2^ intensity were applied to evaluate the photoresponse under each condition. The current ratio between the dark current and the 10th light pulse current was 1.24 A A^−1^ for *V*_g_ = 0 V and 33.7 A A^−1^ for Vg = 7 V, demonstrating an enhanced photoresponse under positive gate bias. In terms of memory characteristics, the post‐illumination current behavior different significantly depending on the gate voltage. At *V*_g_ = 0 V, the current after the 10th light pulse rapidly decayed within 10 s, returning to the dark current. Under *V*_g_ = 7 V, the current after the 10th light pulse exhibited a slight decay and remained almost constant, demonstrating excellent memory performance. To quantitatively compare potentiation behavior for gate bias conditions, the SW was calculated based on the current between the 1st and 10th light pulse, as shown in Figure S9b (Supporting Information). SW can be described by the Equation (2):

(2) $$\mathrm{SW}\;=\frac{A_{10}-A_{1}}{A_{1}}\times 100\%$$

where *A*_1_ and *A*_10_ are the 1st and 10th light pulse current, respectively. The SWs were 59% at *V*_g_ = 7 V and 13% at *V*_g_ = 0 V, indicating a 4.5‐time enhancement under positive gate bias. This significant difference is explained by the charge trapping mechanism illustrated in Figure S9c,d (Supporting Information). Under *V*_g_ = 0 V (Figure S9c, Supporting Information), photoexcited electrons are generated by blue light and a small number of electron become trapped at the DPP‐DTT NW/SiO_2_ interface. Since there is no external voltage to retain the trapped electrons, the device shows poor memory performance. When a *V*_g_ = 7 V is applied (Figure S9d, Supporting Information), the electric field accelerates the transport of photoexcited electrons toward the SiO_2_ surface, leading to a larger number of trapped electrons compared to the *V*_g_ = 0 V. Overall, applying a positive gate bias not only facilitates efficient photoexcited electrons trapping but also develops both the synaptic enhancement and memory characteristics of the device. For depression (Figure 2j), a negative gate bias (*V*_g_ < 0) induces electrostatic repulsion of the trapped electrons, promoting their recombination with holes in the channel and progressively reducing the drain current. This dynamic behavior enables the emulation of biological synapses in a light‐gated, pulse‐programmable architecture.

**Figure 3:**
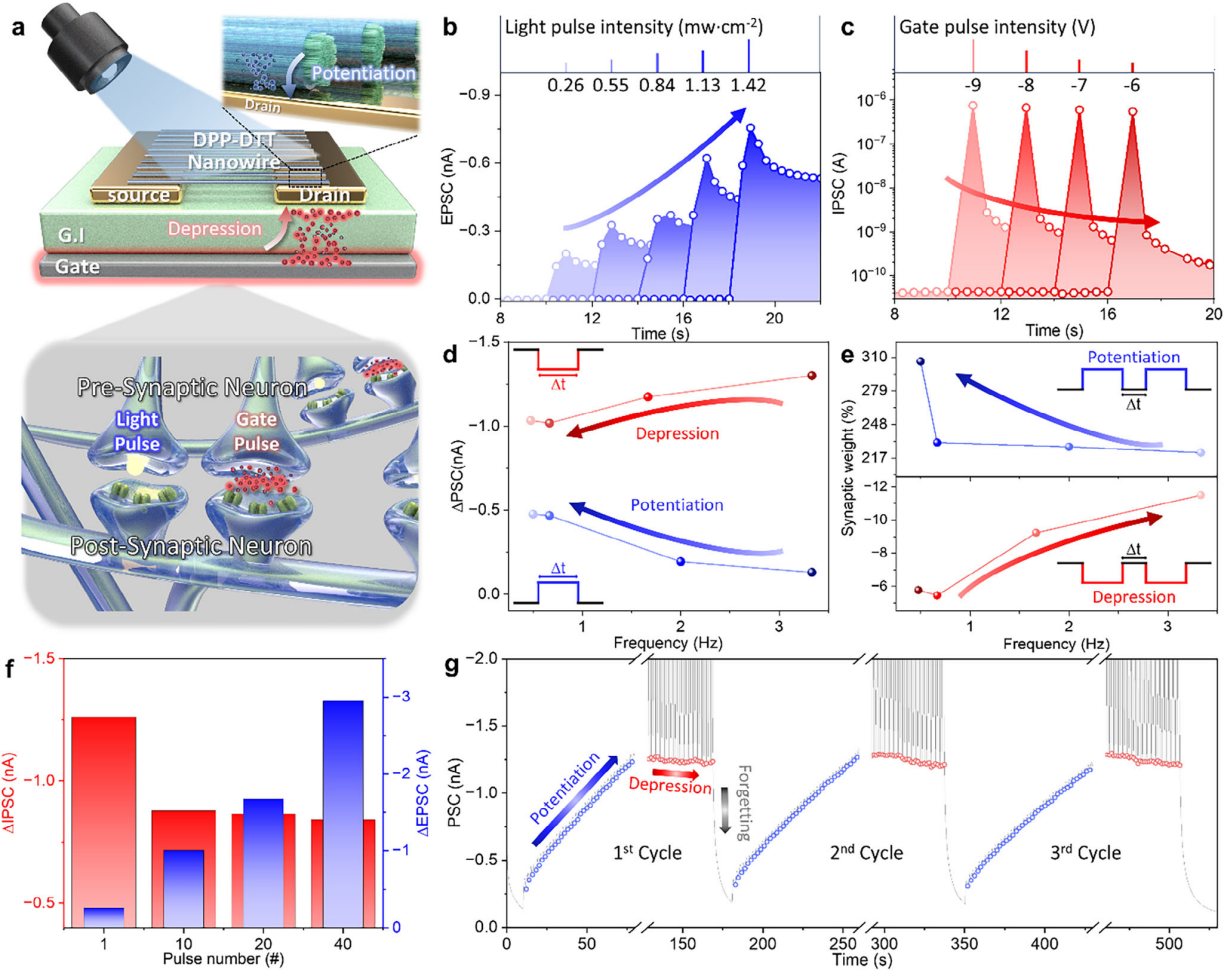
a) Schematic illustration of the structural and functional comparison between a biological synapse and the DPP‐DTT NWs‐based PT. Synaptic plasticity characteristics of the DTT‐DTT NWs‐based PT under varying conditions; b) light pulse intensity (0.26–1.42 mW cm^−2^, 1 s pulse) with a fixed duration of 1 s, c) gate pulse intensity (−6 to −9 V, 0.3 s pulse) with a fixed duration of 0.3 s, d) stimulus duration (0.3–2.1 s) with a light pulse of 0.55 mW cm^−2^ and a gate pulse of −7 V, e) stimulus frequency (0.47–3.33 Hz) with light pulses of 0.55 mW cm^−2^ and gate pulses of −7 V, and f) number of pulses (1–40 cycles) under constant 1 s light pulse and 0.3 gate pulse. g) Potentiation and depression characteristics measured repeatedly for 3 cycles. Potentiation and depression exhibit learning‐forgetting behaviors following each cycle.

The proposed DPP‐DTT NWs‐based PT modulates channel conductivity via photoexcited‐electron trapping at the DPP‐DTT NWs/SiO_2_ interface, enabling its function as an artificial synapse. **Figure** **3**a illustrates the structural and functional analogy between biological and electronic synapses. In biological systems, post‐synaptic current (PSC) is induced by neurotransmitter‐mediated signaling between neurons. Similarly, in the DPP‐DTT NWs‐based PT, a light pulse triggers excitatory PSC (EPSC) for potentiation, while a gate pulse induces inhibitory PSC (IPSC) for depression. Synaptic behavior was first evaluated by varying light intensity and gate bias. As shown in Figure 3b, increasing light intensity (0.26–1.42 mW cm^−2^, 1 s pulse) enhances EPSC, confirming optical potentiation. In Figure 3c, depression was induced by applying negative gate biases from −6 to −9 V under identical pulse conditions. As the negative gate bias reduces, IPSC decreases, reflecting effective synaptic depression. Additionally, Figure S10 (Supporting Information) demonstrates that applying the same gate biases after light‐induced potentiation results in stronger inhibitory behavior, highlighting the effect of prior excitation on synaptic depression. This contrast originates from hole trapping at shallow defect states in DPP‐DTT NWs under strong negative bias, which elevates the subthreshold swing and off‐state current (Figure 2a; Figure S11, Supporting Information).^[^ ^49^, ^50^ ^]^ To validate that positive bias does not influence depression, a constant gate bias of 7 V and periodic 7.025 V pulses were applied (Figure S12, Supporting Information), yielding a stable square‐wave response without IPSC reduction. This confirms that positive bias does not induce hole trapping, whereas excessive negative bias degrades synaptic control. Synaptic plasticity was further modulated by adjusting duration, frequency, and number of stimuli, with fixed light intensity (0.55 mW cm^−2^) and gate bias (−7 V). Figure 3d shows that shorter stimulation duration leads to stronger potentiation (EPSC: −0.13 nA → −4.7 nA) and reduced depression (IPSC: −1.3 nA → −1.03 nA), demonstrating enhanced responsiveness. Frequency‐dependent plasticity was evaluated using ten repeated stimuli and quantified by the SW (Figure 3e). In potentiation, lower stimulus frequency resulted in higher SW (221% → 306%). For depression, higher frequency increased weight from −5.7% to −11.5%. Under fixed intensity and frequency, increasing the number of stimuli from 1 to 40 enhanced EPSC (−0.25 nA → −2.94 nA) and reduced IPSC (−1.26 nA → −0.84 nA), as shown in Figure 3f. In line with Figure 2h, the sustained current state after stimulation indicates a transition from short‐term plasticity (STP) to long‐term plasticity (LTP). The detailed transition characteristics from STP to LTP, depending on pulse duty cycle, frequency, and number, are presented in Figure S13 (Supporting Information). When the stimulation duration was short or the number of pulses was low, the PSC gradually returned to its initial state, exhibiting short‐term plasticity. In all cases, the device demonstrated a successful and consistent transition from STP to LTP, confirming the synaptic capability of the system. To assess device endurance, potentiation and depression were repeated over three cycles (Figure 3g). The PSC was reset after each cycle, maintaining consistent behavior and reflecting a memory‐forgetting characteristic. In neuromorphic computing, the symmetry between potentiation and depression is critical for efficient SW updates. To improve the symmetry of the potentiation‐depression curve, we fixed *V*g pulse of −7 V and its duration with 0.3 s, while adjusting the interval between stimuli from 1 to 2 s. The number of potentiation‐depression cycles was increased from 3 to 50 to evaluate the robustness and endurance of the device. During the repeated 50 cycles stimulation for the potentiation–depression curve, a total of 4000 stimuli were applied, as shown in Figure S14 (Supporting Information). The symmetry between the potentiation and depression curves within a single cycle was significantly improved compared to the cycle presented in Figure 3g. After the 50 cycles, PSC increased from 1.40 to 1.93 nA, corresponding to an increase of 37%. This characteristic is similar to the gradual strengthening of synaptic connections in biological systems. The demonstrated reliability and biologically analogous synaptic behavior highlight the potential of the DPP‐DTT NW‐based PT for machine learning systems requiring short‐term learning and iterative weight updates.

**Figure 4:**
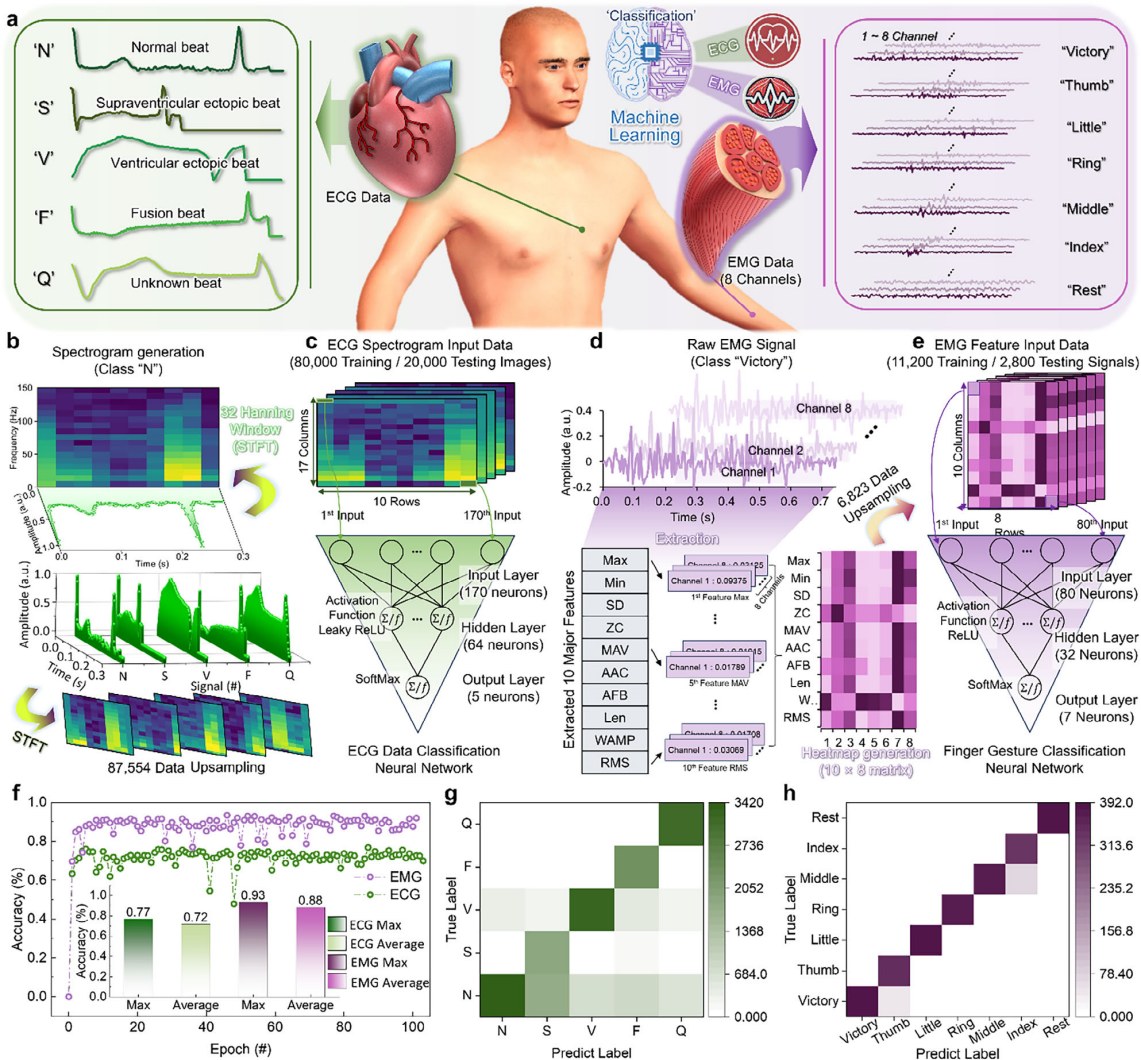
a) Schematic illustration of 5 ECG signal classes and 7 EMG signal classes classification using a neural network based on MLP architecture consisting of DPP‐DTT NWs‐based PT. b) Preprocessing of raw ECG signal (187 samples) from the MIT‐BIH Arrhythmia Database via STFT using a 32‐sample Hanning window and 16‐sample overlap to extract time‐frequency features; generation of 17 × 10 spectrograms representing 5 ECG classes. c) Extracted ECG spectrograms (170 inputs) classified using an MLP (170‐64‐5) with Leaky ReLU and Softmax activations; trained on 80000 samples and tested on 20000 samples. d) Feature extraction from EMG signals recorded by a Myo armband; construction of 10 × 8 feature heatmap using 10 major features per channel for 7 EMG classes. e) Flattened EMG feature heatmaps (80 inputs) classified using an MLP (80‐32‐7) with Leaky ReLU and Softmax activations; network trained on 11200 samples and tested on 2800 samples to classify 7 hand gestures. f) Training accuracy for individual ECG and EMG classifications using the DPP‐DTT NWs‐based PT (inset: Histogram of the maximum and average training accuracy). Confusion matrices for the classification test of g) 5 ECG classes and h) 7 EMG classes.

To evaluate the performance of Al‐based physiological analysis, a preliminary classification of ECG and EMG signals was carried out via a hardware‐based neural network simulation built by the dynamic synaptic characteristics of DPP‐DTT NWs‐based PT. Prior to this task, a more general performance evaluation was conducted using a handwritten digit recognition task based on an artificial neural network (ANN) model with the MNIST dataset (Figure S15a, Supporting Information). This evaluated the data analysis and classification potential of DPP‐DTT NWs‐based PT. During 50 training epochs, the maximum accuracy was 88.9% and the average accuracy achieved was 82.6% (Figure S15b, Supporting Information). The confusion matrix shows that the true and predicted labels represent the input and output values of the handwritten digits, which were mostly accurately classified (Figure S15c, Supporting Information). These promising results indicate that our device is suitable for physiological signal analysis, including EGC and EMG signals. As shown in **Figure** **4**a, ECG signals are medically classified into five categories according to the timing and intensity patterns of electrical signals: normal (N), supraventricular ectopic (S), ventricular ectopic (V), fusion (F), and unknown (Q) beats. Similarly, EMG signals classify based on the temporal electrical signals generated by individuals: the index finger, middle finger, ring finger, little finger, thumb, resting gesture, and victory gesture. The classification of ECG and EMG signals involves extracting parameters through preprocessing based on signal characteristics and spectrogram generation, which are then used for classification of physiological signals with an ANN‐based neural network (Figure 4b–h).

The ECG signals consist of 5 classes (“N”, “S”, “V”, “F”, and “Q”) and are processed using Short‐Time Fourier Transform (STFT) with Hanning window function to generate spectrograms (Figure 4b). The neural network for ECG signal classification is trained using 80000 training and 20000 test ECG spectrogram images (Figure 4c). For EMG signal classification, 10 major features are extracted from the specific signal (e.g., class “victory”) of 8 channels for each EMG signal class of 7 finger gestures (Figure 4d). These 10 features include maximum (Max), minimum (Min), standard deviation (SD), zero crossings (ZC), amplitude change (AAC), amplitude of first burst (AFB), mean absolute value (MAV), length of waveform (Len), willison amplitude (WAMP), and root mean square (RMS). The 10 major feature intensities for 8 channels are transformed into a heatmap represented by a 10 × 8 matrix. In the entire EMG dataset consisting of 7 finger gestures, 11200 training signals and 2800 test signals are used as input for the neural network for EMG signal classification (Figure 4e). Figure 4f shows the training accuracy obtained from designed neural networks for ECG and EMG signal classification. During 100 training epochs, the maximum accuracy is 76.8% (average accuracy is 71.6%) for ECG signal classification and 93.4% (average accuracy is 88.3%) for EMG signal classification. These results are comparable to the maximum accuracy of 88.9% (average accuracy is 82.6%) observed with the MNIST for handwritten digit classification. Furthermore, the confusion matrices for 5 ECG signal classes and 7 EMG signal classes show high contrast in classification accuracy (Figure 4g,h). To improve the classification accuracy for both MNIST and ECG datasets, a convolutional neural network (CNN) architecture optimized for image recognition tasks was adopted (Figure S16a, Supporting Information). The CNN‐based approach improved the classification accuracy for both the ECG spectrogram and MNIST datasets under 50 training epochs (Figure S16b, Supporting Information). For the ECG spectrogram classification, the CNN achieved a maximum accuracy of 89% and an average accuracy of 86.2%, while for the MNIST dataset, it reached a maximum accuracy of 97.4% and an average accuracy of 97.2%. Compared to the Multi‐Layer Perceptron (MLP) network (Figure 4g,h), the confusion matrix results for both datasets demonstrate that the CNN architecture offers enhanced classification performance (Figure S16c,d, Supporting Information), indicating its effectiveness on a variety of image‐based datasets. To further evaluate optical modulation in a biologically relevant context, we conducted a CNN‐based simulation using Canadian Institute for Advanced Research (CIFAR)‐10 datasets, which are widely used for benchmarking vision‐based neural networks. The dataset comprises 50000 training and 10000 test samples across 10 classes of RGB natural images and was used for object classification (airplane, automobile, bird, cat, deer, dog, frog, horse, ship, truck). The object recognition simulation achieved a maximum accuracy of 83.8% and an average accuracy of 82.6% over 50 training epochs, as shown in Figure S17a (Supporting Information). In the confusion matrix, the 10 labels show a close match between the true images and the predictions obtained through CNN training (Figure S17b, Supporting Information). These results demonstrate that the device can support learning and classification of optically encoded visual inputs in a neuromorphic vision system.

To further evaluate the potential of the proposed device, we summarize key comparison parameters such as device type, material, stimuli source, number of conductance states, cycle durability, network model, and recognition accuracy.^[^ ^43^, ^44^, ^51^, ^52^, ^53^, ^54^, ^55^, ^56^, ^57^ ^]^ As summarized in Table S2 (Supporting Information), our DPP‐DTT NW‐based PT demonstrated a high classification accuracy of 97.4% for MNIST, 93.4% for EMG, 89.0% for ECG, and 83.8% for CIFAR‐10 using various neural network models including CNN and MLP. These results are either superior to or comparable with those reported in previous studies using electrical or hybrid stimulation. In Table S3 (Supporting Information), the synaptic performance of various DPP‐DTT‐based devices is compared under different structural and optical configurations,^[^ ^43^, ^44^, ^56^, ^57^ ^]^ suggesting that UV‐dependent strategies often involve complex material systems and face stability concerns due to prolonged UV exposure. Our device adopts a structurally simplified architecture based solely on pristine DPP‐DTT NWs and operates under visible light illumination, eliminating the need for additional charge‐trapping layers or UV excitation. Furthermore, the proposed device exhibits stable operation and achieves high recognition accuracy with a small number of stimuli. This performance highlights that the optically modulated synapse in our device is suited for a wide range of classification tasks, including image and biosignal recognition, while offering advantages in fabrication simplicity, energy efficiency and high‐speed learning.

## 3 Conclusion

We successfully engineered neuromorphic computing for classifying various physiological signals, such as ECG and EMG signals, by implementing technologies ranging from organic semiconductor polymer patterning to synaptic devices and AI algorithm functions. The NW‐structured patterned DPP‐DTT enables reproducible nanoscale fabrication and enhances the photogating effect by increasing the probability of photoecited‐electron trapping due to its high surface‐to‐volum ratio. The presence of OH^−^ defects at the DPP‐DTT NWs/SiO_2_ interface of the proposed DPP‐DTT NWs‐based PT causes trap‐detrap according to optical and electrical stimuli, mimicking distinct synaptic behaviors such as potentiation and depression. Notably, the synaptic behavior of the DPP‐DTT NWs device was modulated by PSC depending on parameters such as stimulus intensity (light pulse: 0.26–1.42 mW cm^−2^, gate pulse: −6 to −9 V), duration (0.3–2.1 s), frequency (0.47–3.33 Hz), and number of times (1–40 cycles). The DPP‐DTT NWs‐based PT mimics the learning‐forgetting characteristics of synapse in the human brain. The capability of our synaptic device achieved 97.4% accuracy for a handwritten digit recognition simulation based on ANN learning using the MNIST. Furthermore, it obtained 89.0% accuracy for classifying 5 ECG signal classes, 93.4% accuracy for classifying 7 EMG signal classes, and 83.8% accuracy for object recognition using CIFAR‐10, demonstrating performance comparable to handwritten digit recognition. These results suggest that the neural network based DPP‐DTT NWs synaptic device opens up potential applications in neuro‐inspired vision systems and the biohealthcare field for classifying and diagnosing various human physiological signals.

## 4 Experimental Section

### Preparation of Stamp and DPP‐DTT Solution

The PDMS stamp was fabricated using a template produced by photolithography. The groove of template is a width of 10 µm and a depth of 1.2 µm. The PDMS solution was prepared by mixing the pre‐polymer and curing agent in a 10:1 ratio (Sylgard 184, Dow Corning Co., Midland, MI, USA). This mixture was cast on the template and dried at 60 °C. After drying, the PDMS was peeled off from the template, resulting in a stamp that replicates the morphology of the template. The DPP‐DTT (Ossila, Mw = 203956, PDI = 3.09) powder was dissolved in DCB (Sigma‐Aldrich, St. Louis, MO, USA) with constant stirring at 70 °C for 24 h.

### Fabrication of DPP‐DTT NW‐Based Synaptic Device

The SiO_2_/Si substate was cleaned by ultrasonication with acetone, isopropanol, and deionized water for 10 min. The cleaned substrate was dried using a N_2_ blowing gun. The thickness of SiO_2_ is 100 nm and the heavily doped‐Si utilized as a back gate. The Au/Ti (40 nm, 10 nm) electrodes were deposited using an E‐beam evaporator after patterning by photolithography on the substate. The length between the electrodes is 10 µm. The 1 µL of DPP‐DTT solution was dropped on the substate, upon which the PDMS stamp was carefully laminated under ambient conditions. Annealing was performed at 140 °C for 4 h to remove residual solvent. For comparison of the optoelectronic properties with the DPP‐DTT NW structure, a DPP‐DTT thin film was spin‐coated at 2500 rpm for 60 s and subsequently annealed at 140 °C for 4 h. To evaluate the DPP‐DTT NWs formation depending on SiO_2_ surface treatment, we modified the surface to be hydrophilic via O_2_ plasma treatment prior to the growth of DPP‐DTT NWs, as shown in Figure S18a (Supporting Information). The hydrophilic surface induced by O_2_ plasma strongly attracted the DCB solvent, thereby inhibiting the capillary force between sidewalls of the PDMS and SiO_2_ surface. As shown in Figure S18b (Supporting Information), the intended NW alignment resulted in discontinuous dot‐like formations rather than forming continuous NWs. This result interrupted the formation of the conductive channel between the drain and source electrodes.

### Characterization of DPP‐DTT NW Film and DPP‐DTT NW‐Based Synaptic Device

The DPP‐DTT NWs morphology was analyzed using SEM (CLARA, Tescam, Brno, Czech), EDS (CLARA, Tescam, Brno, Czech), AFM (PSIA XE‐100, Park Systems, South Korea). The absorbance of the DPP‐DTT NWs was measured by UV‐Vis spectroscopy (JASCO 770, Jasco Inc., USA). XPS (Kratos AXIS Nova, Kraos Analytical Ltd., UK) and contact angle of 1 µL DI water droplet (Contact Angle Goniometer, Ossila Ltd., UK) were used to prove that DCB‐treated SiO_2_ surface had fewer OH^−^ groups than pristine SiO_2_. O_2_ plasma treatment of the SiO_2_ surface was carried out at 20 sccm, 100 W, and 50 Hz for 30 min (CIONE 4, Femtoscience Inc., South Korea). A Keithley 4200A‐SCS and a probe station were used under ambient conditions to measure the current‐voltage, the current‐time characteristics, synaptic performance, and endurance of the DPP‐DTT NWs‐based PT. For photoresponsivity calculations, the active areas of the DPP‐DTT NW and thin‐film devices were defined as 8.46 × 10^−6^ cm^2^ and 2.5 × 10^−4^ cm^2^, respectively. The 455 (blue), 530 (green), and 660 nm (red) light pulses were generated using the conventional LED light sources (Mounted LEDs, Thorlabs Inc., New Jersey, USA) to stimulate synaptic behavior of the DPP‐DTT NWs‐based PT.

### Neural Network Architecture and SW Update

A simulation was performed to evaluate the computational performance of a neural network based on an ANN implemented using the DPP‐DTT NWs‐based PT. The neural network was designed based on a MLP architecture consisting of an input layer, hidden layer, and output layer. CNN architecture employs a pre‐trained Visual Geometry Group 16 (VGG16) network as a feature extractor. It consists of five convolutional blocks (Conv1–Conv5), each followed by 2 × 2 max‐pooling layers to reduce spatial resolution. Each block includes two or three convolutional layers with 3 × 3 kernels and a stride of 1. SW can be updated by adjusting the pair of conductance states (*G* ^+^ and *G* ^−^) through potentiation or depression. The weight is encoded by the difference between the conductance of two artificial synapses (*W* = *G* ^+^ and *G* ^−^). During training, SWs are updated by increasing either *G* ^+^ or *G* ^−^, depending on the weight change (*ΔW*) determined from the loss function. When the weight is potentiated, *G* ^+^ was increased while *G* ^−^ remained fixed. Conversely, when the weight was depressed, *G* ^−^ was increased with *G* ^+^ held constant. This approach reflects the unidirectional nature of conductance modulation, allowing for effective implementation of weight changes.

### MNIST Handwritten Digits Simulation

The MNIST dataset consists of 28 × 28 pixel grayscale images, each of which is flattened into 784 input vectors. These vectors are applied to the input layer of a MLP with an architecture comprising an input layer (784 neurons), hidden layer (64 neurons), and output layer (10 neurons). Rectified Linear Unit (ReLU) activation function is used in the hidden layer, and a Softmax function is applied to the output layer. For the CNN‐based MNIST classification, handwritten digit images were resized to 224 × 224 × 3 by duplicating the single channel to match the input requirements of the VGG16 architecture. The network architecture consists of five convolutional blocks (Conv1–Conv5), where Conv1 and Conv2 include two convolutional layers each, and Conv3 to Conv5 comprise three layers per block. All convolutional layers use 3 × 3 kernels with a stride of 1, followed by 2 × 2 max‐pooling layers to progressively reduce spatial resolution. The final 7 × 7 × 512 feature map is flattened and fed into two fully connected layers with 128 and 64 neurons, respectively, and an output layer with 10 neurons corresponding to the digit classes. The dataset contains 60000 training images and 10000 test images, with each output neuron corresponding to one of the 10 digit classes (0–9).

### ECG Classification Simulation

The ECG signals were obtained from the raw data provided by the MIT‐BIH Arrhythmia Database, with each signal consisting of 187 samples. To extract time‐frequency features, STFT was applied using a Hanning window (window size is 32, overlap is 16). The resulting spectrogram was converted into an image of size 17 × 10, which was flattened into a 170 input vectors for the MLP. The neural network was structured with an input layer (170 neurons), a hidden layer (64 neurons), and an output layer (5 neurons). Leaky ReLU and Softmax activation functions were applied to the hidden and output layers, respectively. For the CNN‐based ECG classification simulation, ECG spectrogram images were resized to 224 × 224 × 3 to meet the input requirements of the VGG16 architecture. These images were processed through five convolutional blocks (Conv1–Conv5), each consisting of two or three convolutional layers with 3 × 3 kernels and a stride of 1, followed by 2 × 2 max‐pooling layers to reduce spatial resolution. The resulting 7 × 7 × 512 feature map is flattened and passed through two fully connected layers with 128 and 64 neurons, respectively. The dataset consisted of 80000 training samples and 20000 test samples. The output layer was designed to classify five heartbeat categories: N, S, V, F, and QMulti‐Layer Perceptron.

### EMG Classification Simulation

The EMG signals were obtained from a publicly available dataset recorded using the Myo armband. Each signal consisted of 150 samples acquired from 8 channels. The dataset provides 10 pre‐extracted major features for per channel, including maximum, minimum, standard deviation, zero crossings, amplitude change, amplitude of first burst, mean absolute value, length of waveform, willison amplitude, and root mean square. These features were arranged into a 10 × 8 feature heatmap, which was flattened into an 80 input vectors for the MLP. The network architecture comprised an input layer (80 neurons), a hidden layer (32 neurons), and an output layer (7 neurons). Leaky ReLU and Softmax activation functions were applied to the hidden and output layers, respectively. The dataset consisted of 11200 training samples and 2800 test samples. The output layer was designed to classify seven hand gestures: index finger, middle finger, ring finger, little finger, thumb, resting gesture, and victory gesture.

### CIFAR‐10 Object Classification Simulation

The CIFAR‐10 dataset consists of 32 × 32‐pixel RGB images, which are resized to 224 × 224 × 3 to meet the input requirements of the VGG16‐based CNN architecture. These images are passed through five convolutional blocks, each containing two or three convolutional layers with 3 × 3 kernels and a stride of 1, followed by 2 × 2 max‐pooling layers for spatial resolution. The final 7 × 7 × 512 feature map is flattened into a 25088‐D vector and applied to the fully connected layers comprising 128 and 64 neurons, respectively, followed by an output layer with 10 neurons corresponding to the image classes. Leaky ReLU and Softmax activation functions are used in the hidden and output layers, respectively. The dataset consisted of 50000 training images and 10000 test images. The output layer was designed to classify ten object categories: airplane, automobile, bird, cat, deer, dog, frog, horse, ship, truck.

## Conflict of Interest

The authors declare no conflict of interest.

## Supporting information



Supporting Information

## Data Availability

The data that support the findings of this study are available from the corresponding author upon reasonable request.
