# Supplementary material for: DPP‐DTT Nanowire Phototransistors for Optoelectronic Synapses in EMG and ECG Signal Classification
Source: Small. 2025 Aug 8;21(48):e06440. doi: 10.1002/smll.202506440 (PMC12674097; doi:10.1002/smll.202506440)
Supplement: Supplementary file 1 — Supporting Information [file SMLL-21-e06440-s001.docx]

Supporting Information

DPP-DTT Nanowire Phototransistors for Optoelectronic Synapses in EMG and ECG Signal Classification

*Wangmyung Choi^1^, Jin Seok Yoon^2^, Won Woo Lee^3^, Gun Ho Hong^4^, Hyeonjung Kim^4^,*

*Seyong Oh^4,*^, Young Tea Chun^2,*^, and Hocheon Yoo^1,*^*

^1^Department of Electronic Engineering, Hanyang University, 222 Wangsimni-ro, Seoul, 04763, Republic of Korea

^2^Division of Electronics and Electrical Information Engineering, Korea Maritime and Ocean University, 727 Taejong-ro, Busan 49112, Republic of Korea

^3^Department of Artificial Intelligence Semiconductor Engineering, Hanyang University, 222 Wangsimni-ro, Seoul, 04763, Republic of Korea

^4^Division of Electrical Engineering, Hanyang University ERICA, 55 Hanyangdaehak-ro, Ansan 15588, Republic of Korea

*Correspondence should be addressed to: seyongoh89@hanyang.ac.kr (S. O.), ytc24@kmou.ac.kr (Y. T. C.), hocheon@hanyang.ac.kr (H. Y.)

W. Choi, J. S. Yoon, W. W. Lee, and G. H. Hong contributed equally to this work

Keywords: DPP-DTT nanowire; Phototransistor; Photo-gating effect; Neuromorphic device; Physiological signal classification; Electrocardiogram; Electromyography;

**Table of contents**

**Figure S1.** Schematic illustration of the fabrication process for DPP-DTT NW patterns using soft lithography stamping.

**Figure S2.** SEM images and EDS line-scan profiles demonstrating the formation of DPP-DTT NWs without residual polymer.

**Figure S3.** Cross-sectional SEM image of the DPP-DTT NWs showing groove region.

**Figure S4.** Threshold voltage of the DPP-DTT NWs-based PT in various measurement conditions.

**Figure S5.** Contact angle images and surface XPS analysis of the SiO_2_ surface before and after DCB treatment.

**Figure S6.** Comparison of the hysteresis characteristics between DPP-DTT-based film and NW devices.

**Figure S7.** Schematic illustration of the synaptic potentiation mechanism induced by light stimulation.

**Figure S8.** Comparison of the photo-gated threshold voltage shift between DPP-DTT-based film and NW devices.

**Figure S9.** Comparison of synaptic potentiation behavior at gate biases of 0 V and 7 V.

**Figure S10.** Synaptic depression behavior and SW after potentiation by light stimuls.

**Figure S11.** Comparison of subthreshold swing values in hysteresis characteristics.

**Figure S12.** The current–time characteristic analysis indicating the absence of depression behavior under constant positive gate bias pulse.

**Figure S13.** STP-to-LTP transition under different duty cycles, frequencies, and numbers of light pulses.

**Figure S14.** Synaptic endurance characteristics over 50 repeated potentiation–depression cycles.

**Figure S15.** MNIST handwritten digit classification result using DPP-DTT NWs-based PT.

**Figure S16.** ECG and EMG signal prediction results using a convolutional neural network (CNN) model.

**Figure S17.** CIFAR-10 classification result using CNN model.

**Figure S18.** OM image of DPP-DTT NWs formed after O_2_ plasma treatment of the SiO₂ surface.

**Table S1.** Summary of trap-dependent parameters comparing thin-film and NW devices.

**Table S2.** Summary of synaptic device with optical and electrical stimuli.

**Table S3.** Summary of synaptic performance of DPP-DTT devices under different structural and optical conditions.


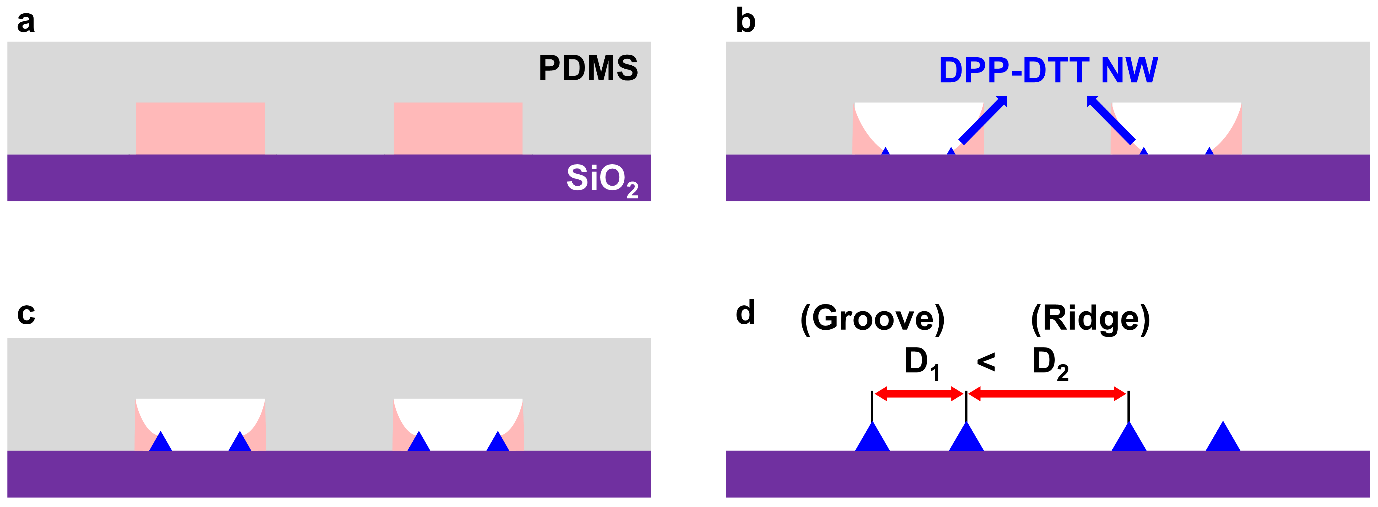


**Figure S1.** Schematic illustration of the 1D DPP-DTT NWs pattern formation process. (a) The solution is stamped using a PDMS mold. (b) During solvent drying, DPP-DTT NW seeds form along the groove edges due to capillary effects. (c) Over time, the NWs grow into triangular shapes. (d) After the solvent evaporates, removal of the PDMS mold yields a patterned structure in which the NW spacing in the ridge region (D_2_) is larger than that in the groove region (D_1_).


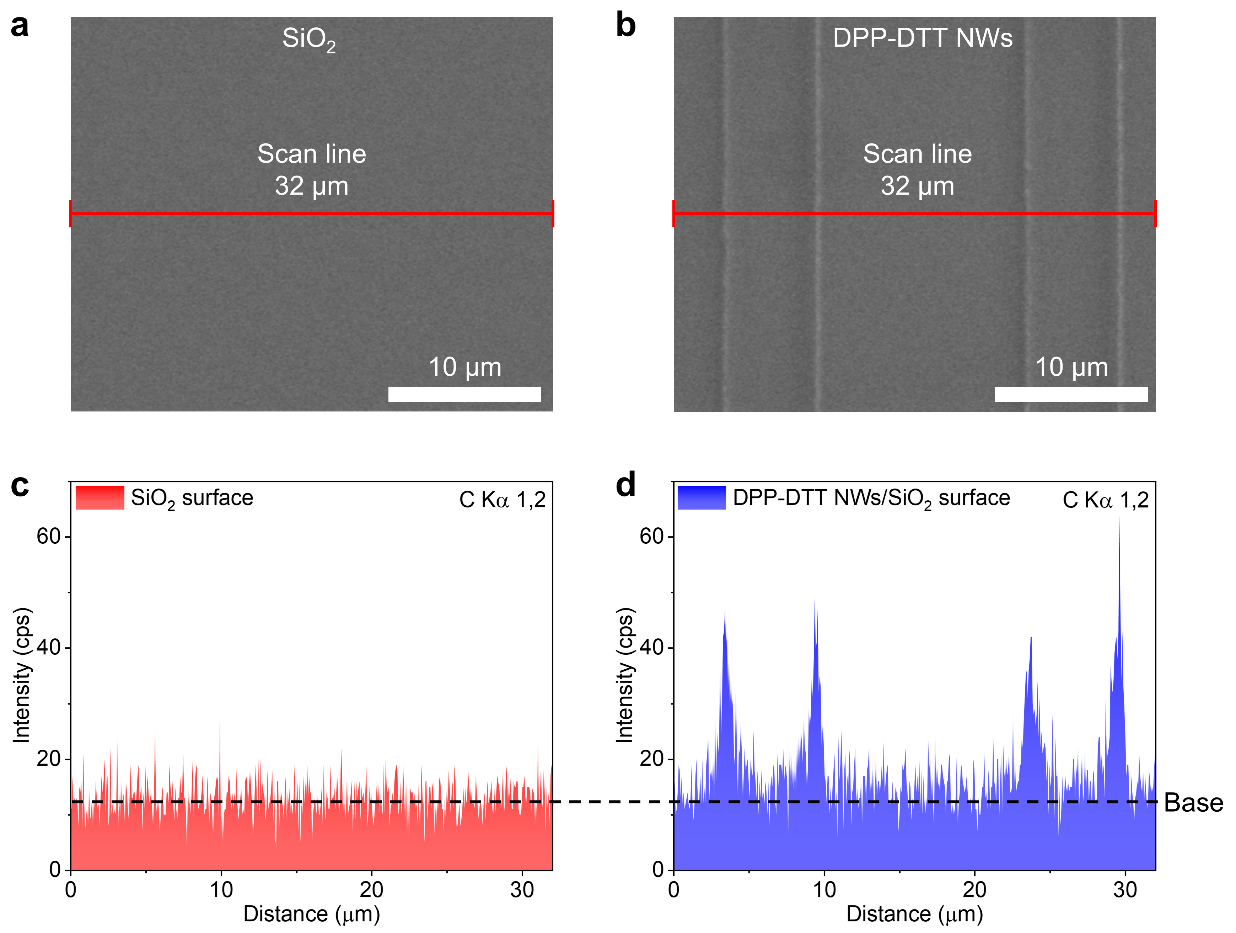


**Figure S2.** SEM surface images of (a) bare SiO_2_ and (b) DPP-DTT NWs (scale bar: 10 μm). EDS line scan profiles of (c) bare SiO_2_ and (d) DPP-DTT NWs, showing the spatial distribution of carbon (C Kα 1,2) along a 32 μm scan line.


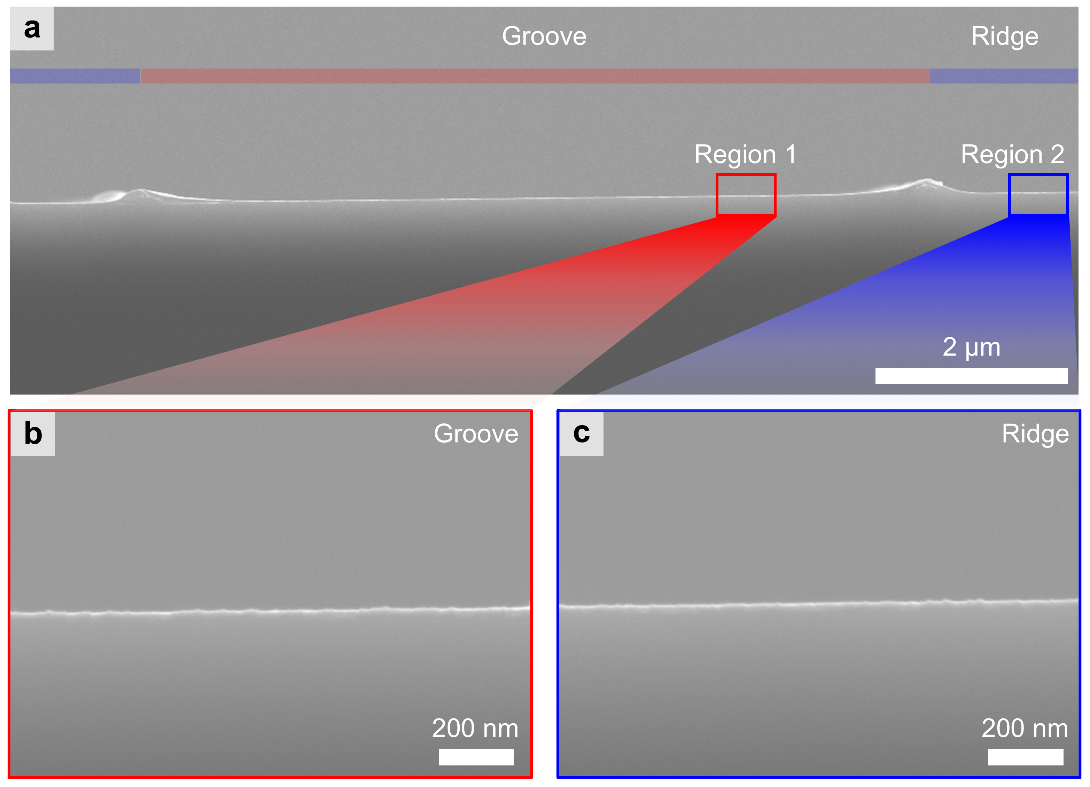


**Figure S3.** (a) SEM Cross section image of DPP-DTT NWs formed in the groove region. SEM Cross section images of (b) groove (Region 1) and (c) ridge (Region 2), respectively, verifying the absence of residual polymer.


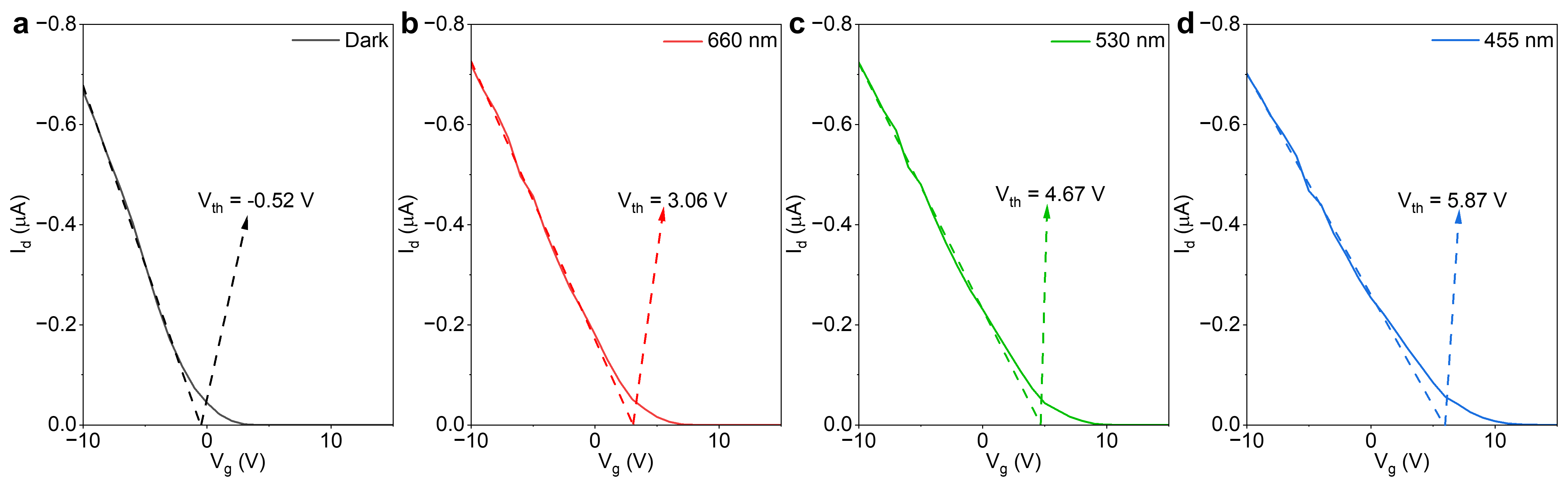


**Figure S4.** Extracted threshold voltage of the DPP-DTT NW-based PT under the different measurement conditions at a fixed intensity (0.55 mW$\cdot$cm^−2^); (a) dark, (b) 660 nm (c) 530 nm (d) 455 nm. In the dark, the threshold voltage is −0.52 V. Upon illumination, a positive shift in threshold voltage is observed due to the photogating effect, with values of 3.06 V, 4.67 V, and 5.87 V for 660 nm, 530 nm, and 455 nm, respectively. The shift becomes more pronounced with decreasing wavelength, indicating enhanced photo-induced charge trapping and modulation of the channel conductivity.


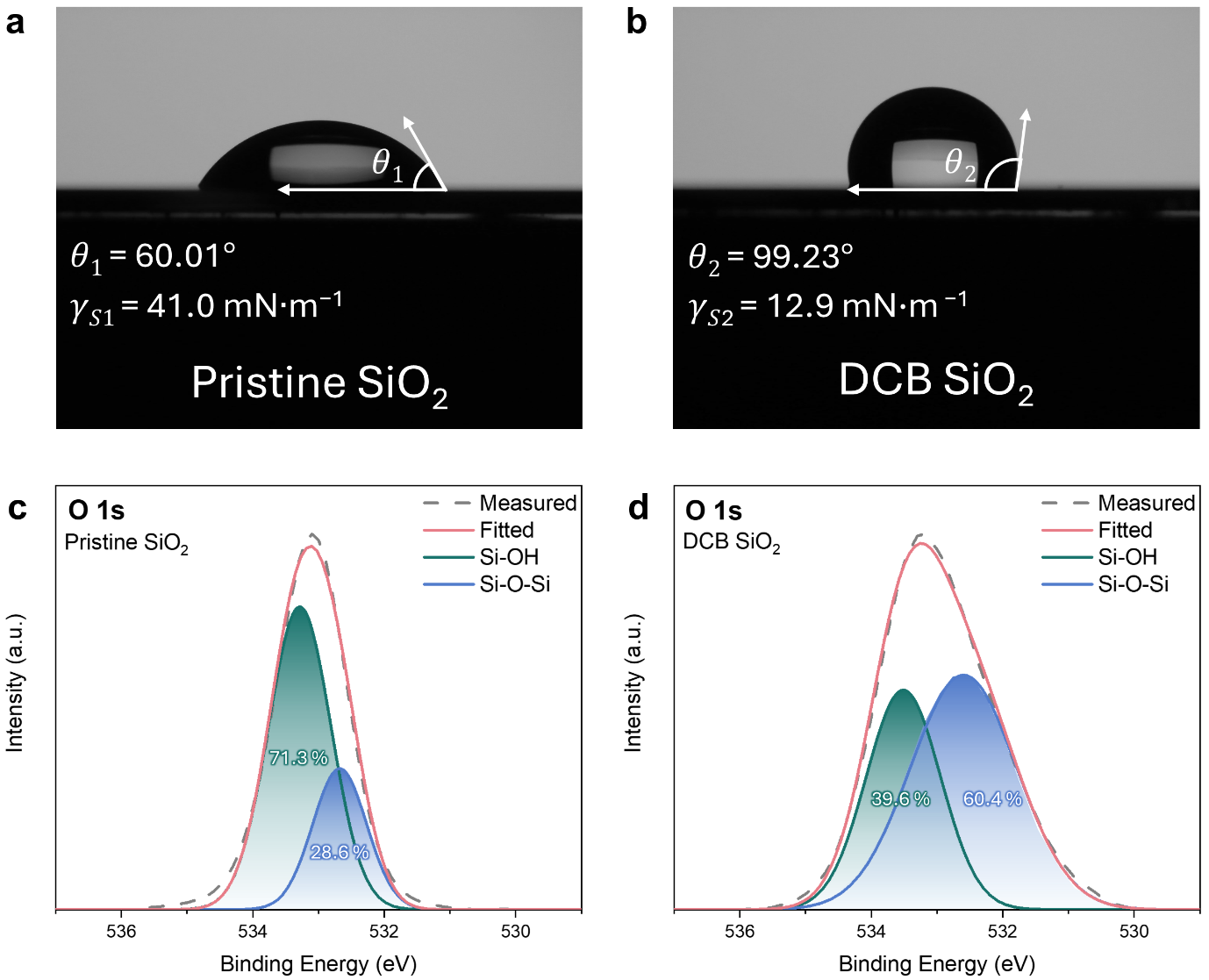


**Figure S5.** Water contact angle image on (a) pristine SiO_2_ and (b) DCB-treated SiO_2_ surface using DI water as the probe liquid. The contact angle increased from 60.01° to 99.23° after interfacial treatment with 1,2-dichlorobenzene, indicating a transition from a hydrophilic to a hydrophobic surface. The corresponding surface energies (γₛ) were calculated as 41.0 mN∙m^−1^ for pristine SiO_2_ and 12.9 mN∙m^−1^ for DCB-treated SiO_2_. XPS profile of (c) pristine SiO_2_ thin film and (d) DCB treated SiO_2_ at the O 1s spectra. The O 1s spectrum was deconvoluted into two peaks at 532.6 eV and 533.5 eV, corresponding to the Si–O–Si and Si–OH bonding, respectively. The Si–OH component on the SiO_2_ surface decreased from 71.3% to 39.6% after treatment with DCB.

**
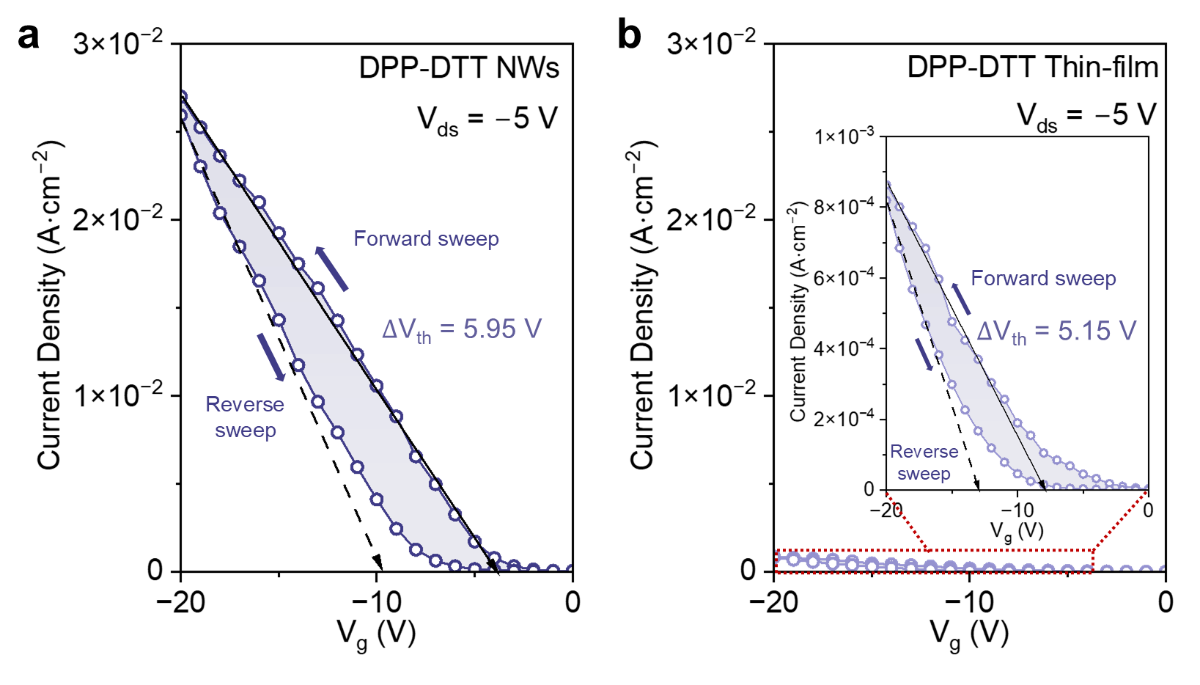
**

**Figure S6.** (a) Hysteresis observed in the transfer characteristics of the DPP-DTT PT with a NW, and (b) a thin-film (inset: enlarged scale graph).


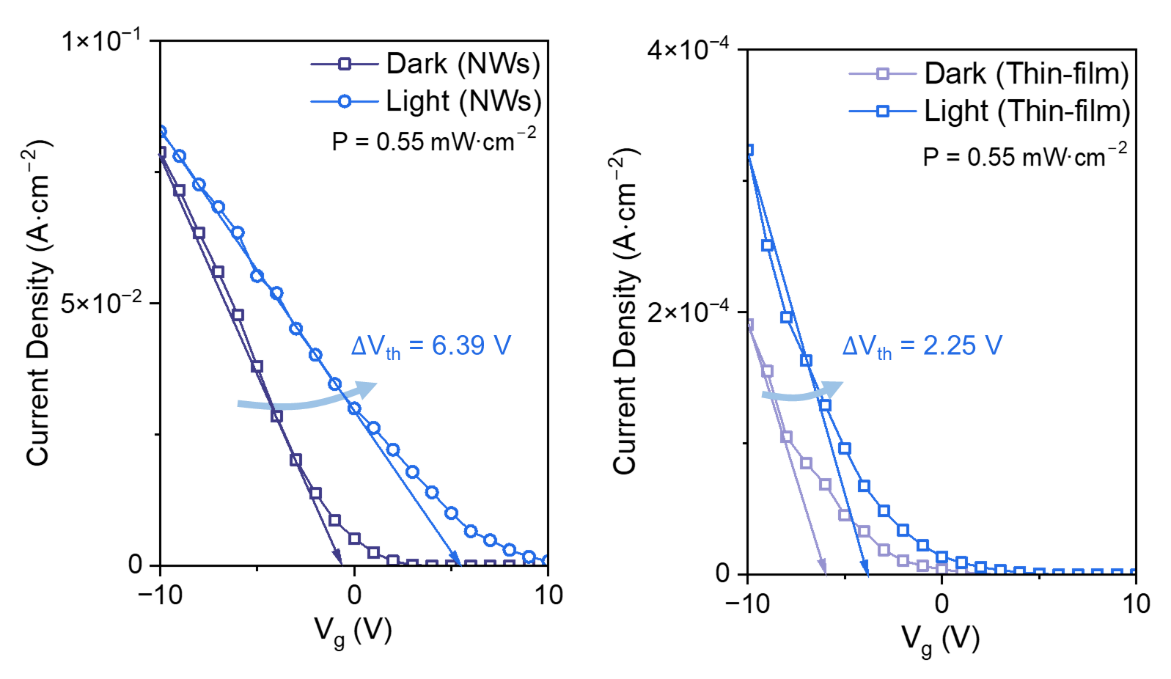


**Figure S7.** (a) Photo-gated threshold voltage shift extracted from the transfer curves (current density-gate voltage) of the NW-based device under 455 nm light at 0.55 mW**∙**cm**^−2^**, showing a *V*_th_ shift of 6.39 V; (b) result for the thin-film device, with a *V*_th_ shift of 2.25 V.


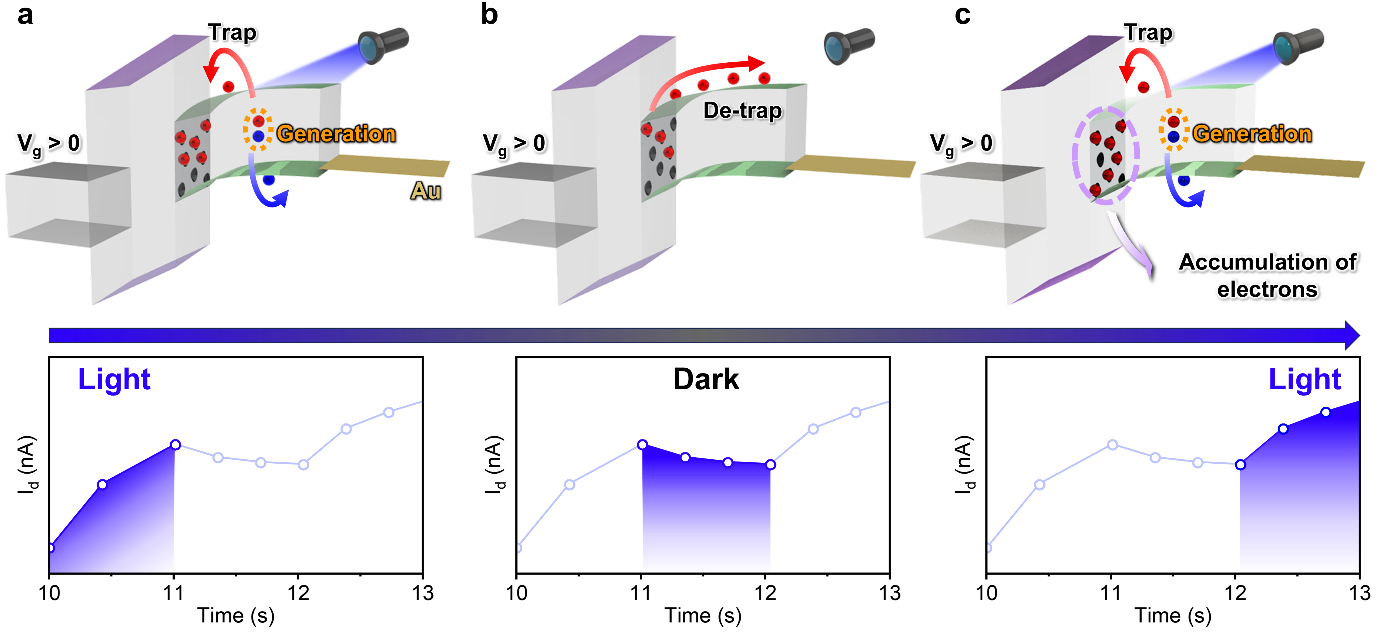


**Figure S8.** Synaptic potentiation mechanism according to the stimulation sequence: (a) initial light exposure induces an increase in drain current; (b) turning off the light leads to partial detrapping and a slight current decrease; (c) electron accumulation through subsequent illumination.


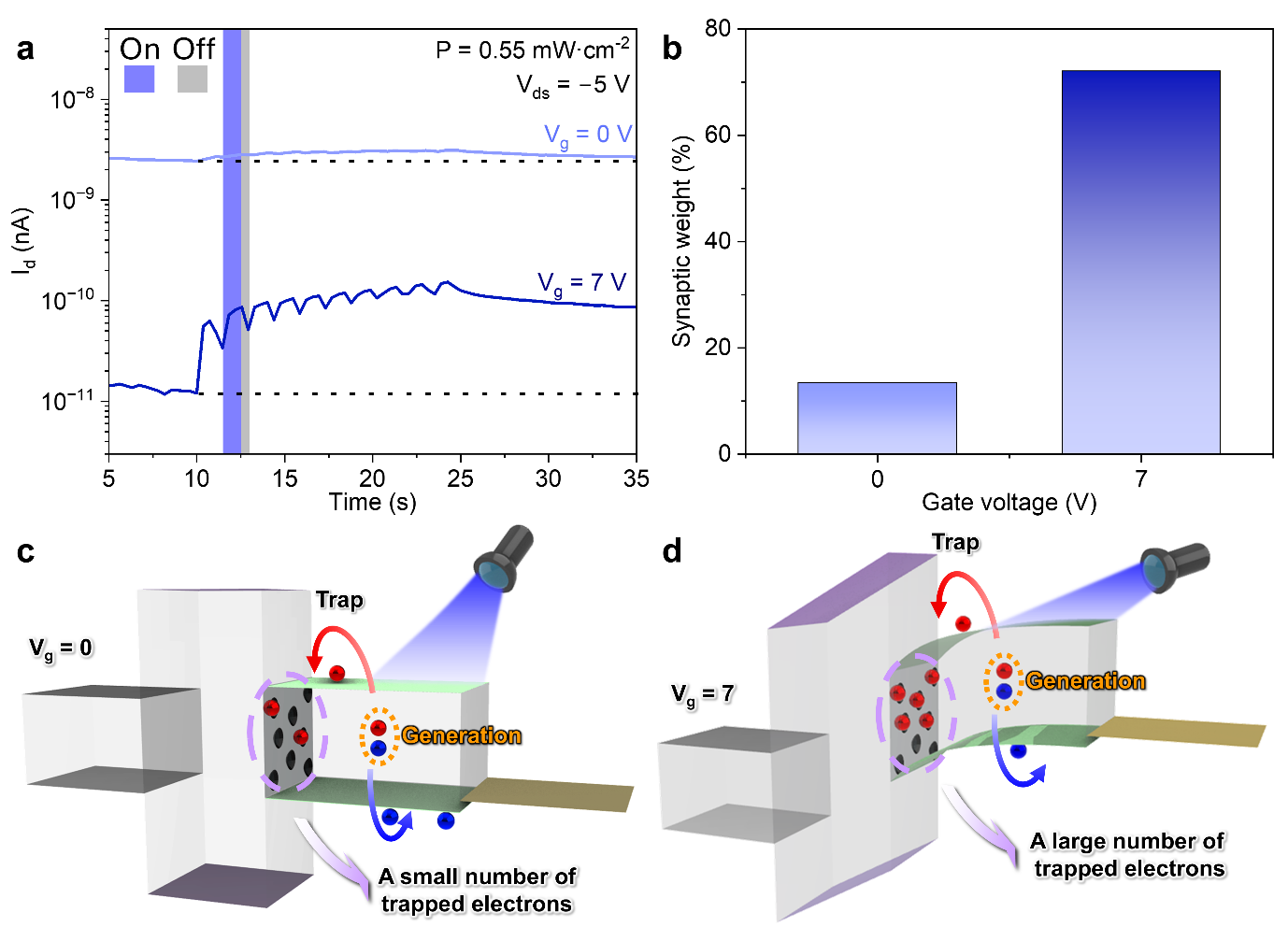


**Figure S9.** (a) Synaptic potentiation of the DPP-DTT NWs-based PT under stimulation by ten light pulse at 455 nm. (b) Calculated synaptic weight under *V*_g_ = 0 V and *V*_g_ = 7 V conditions in Figure S9a. Mechanism of potentiation behavior under (c) *V*_g_ = 0 V and (d) *V*_g_ = 7 V conditions.


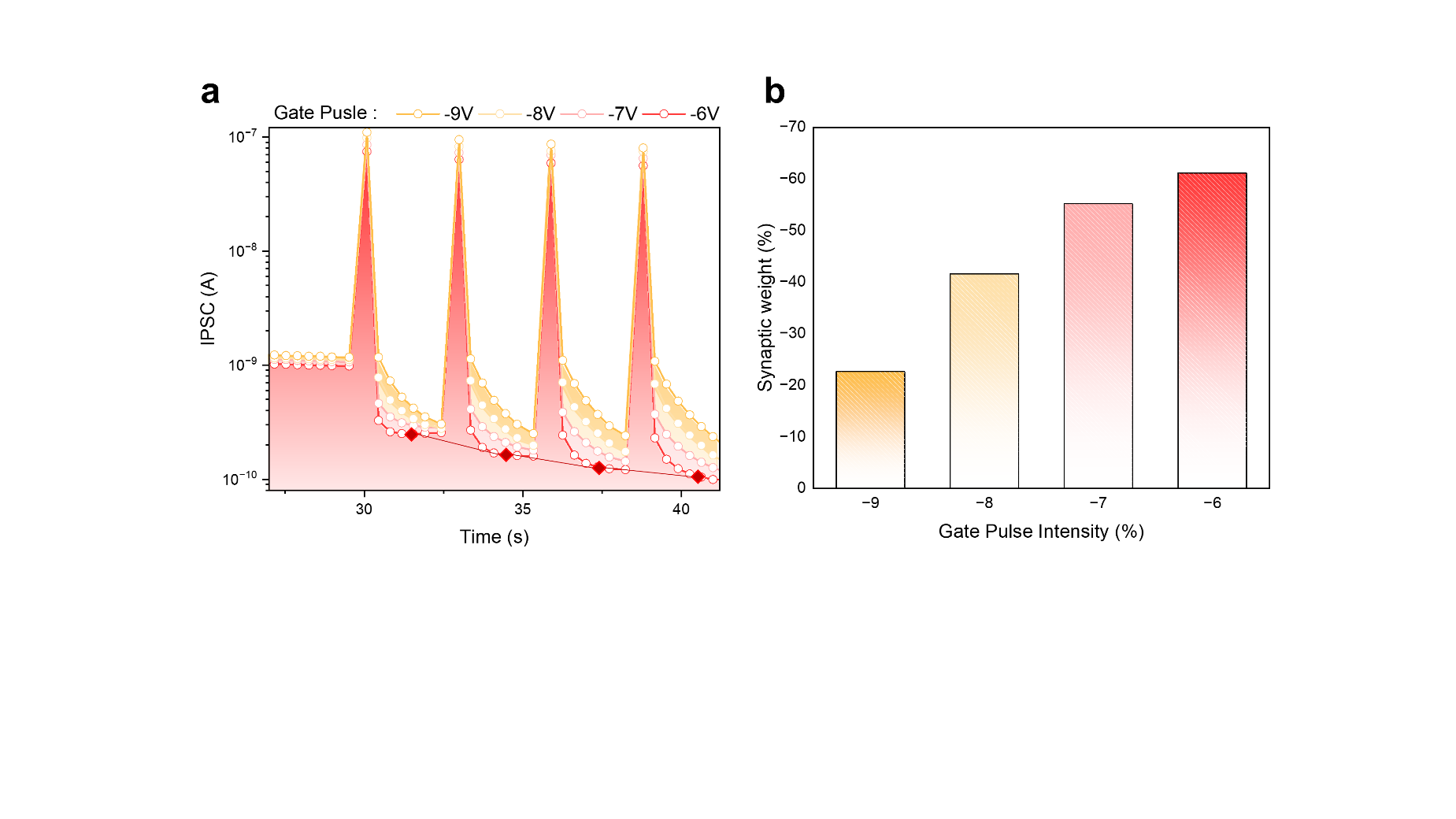


**Figure S10.** Synaptic depression of the DPP-DTT NWs-based PT after potentiation by light stimulus. (a) IPSC profiles under a fixed light pulse (0.55 mW∙cm^−2^, 1 s) followed by gate biases of −9, −8, −7, and −6 V. (b) SW changes of −23%, −42%, −55%, and −61%, respectively, demonstrating progressive depression with increasing gate bias.


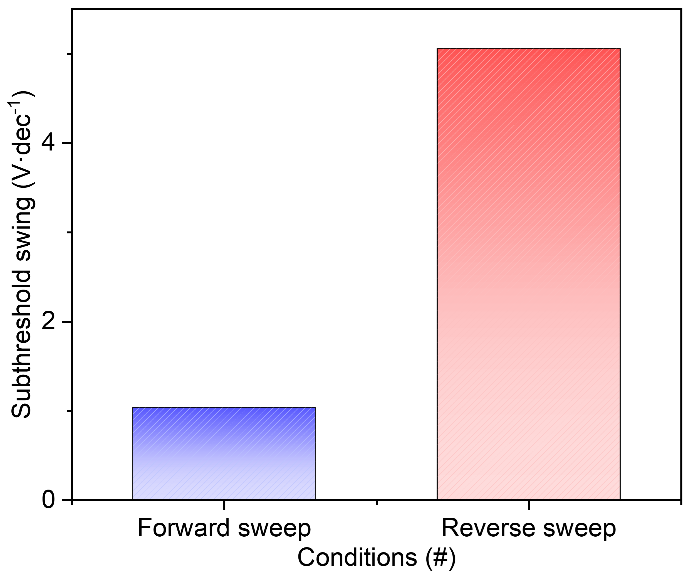


**Figure S11.** Comparison of subthreshold swing values for the DPP-DTT NWs-based PT under reverse and forward gate voltage sweeps. The increase in subthreshold swing values from 1.04 V·dec^−1^ in the forward sweep to 5.06 V·dec^−1^ in the reverse sweep indicates an increase in hole trapping and hysteresis under negative gate bias conditions.


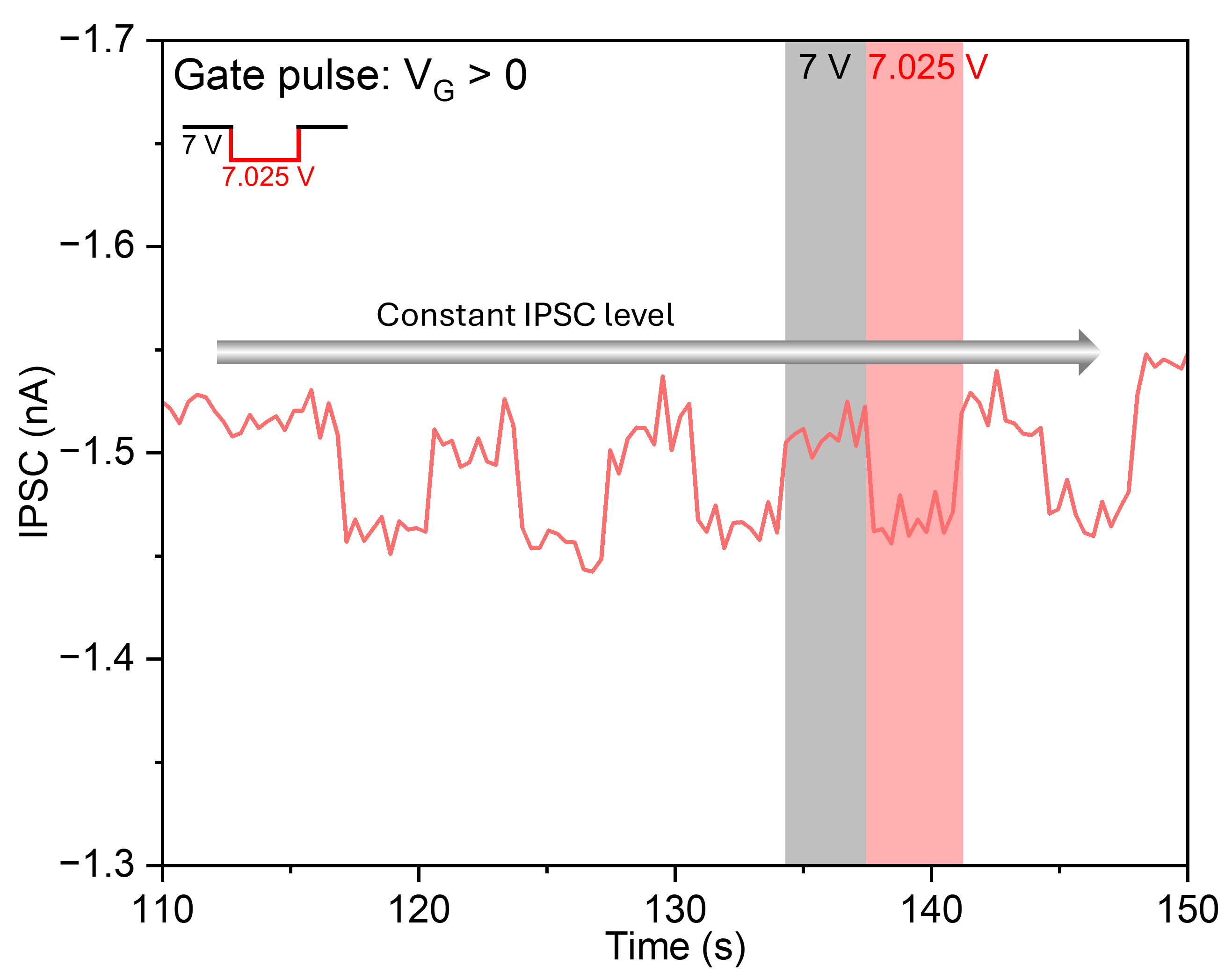


**Figure S12.** Current-time characteristic under periodic 7.025 V gate pulses with a constant gate bias of 7 V. Stable IPSC level without reduction, indicating no hole trapping or depression behavior under positive gate bias.


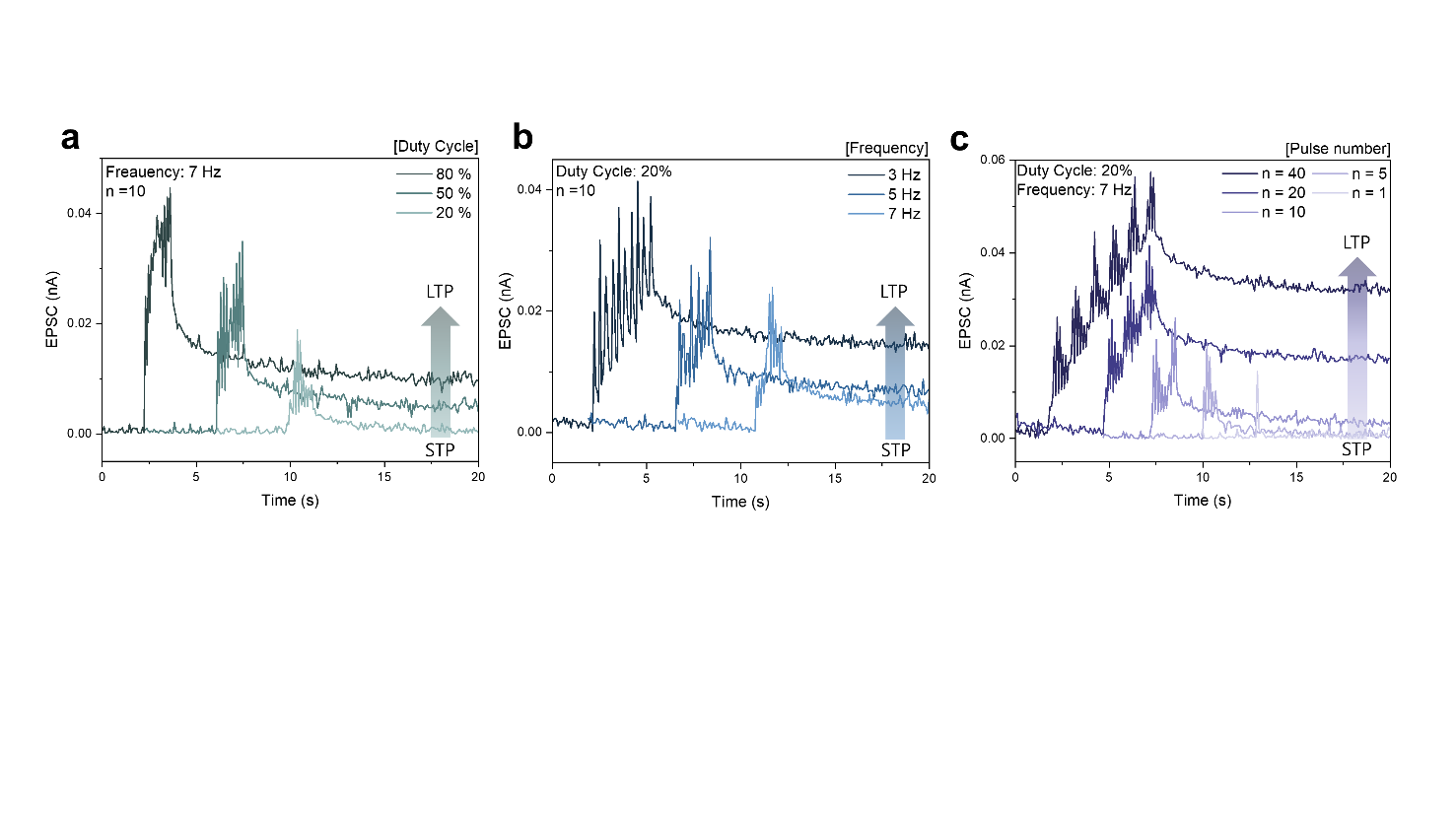


**Figure S13.** STP-to-LTP transition characteristics under varying light pulse (0.55 mW·cm^−2^) conditions; (a) Varying duty cycle of the light pulse (20%, 50%, 80%) under fixed frequency (7 Hz) and pulse number (n = 10). (b) Varying frequency of the light pulse (3, 5, 7 Hz) under fixed duty cycle (20%) and pulse number (n = 10). (c) Varying pulse number (n = 1, 5, 10, 20, 40) under fixed duty cycle (20%) and frequency (7 Hz).


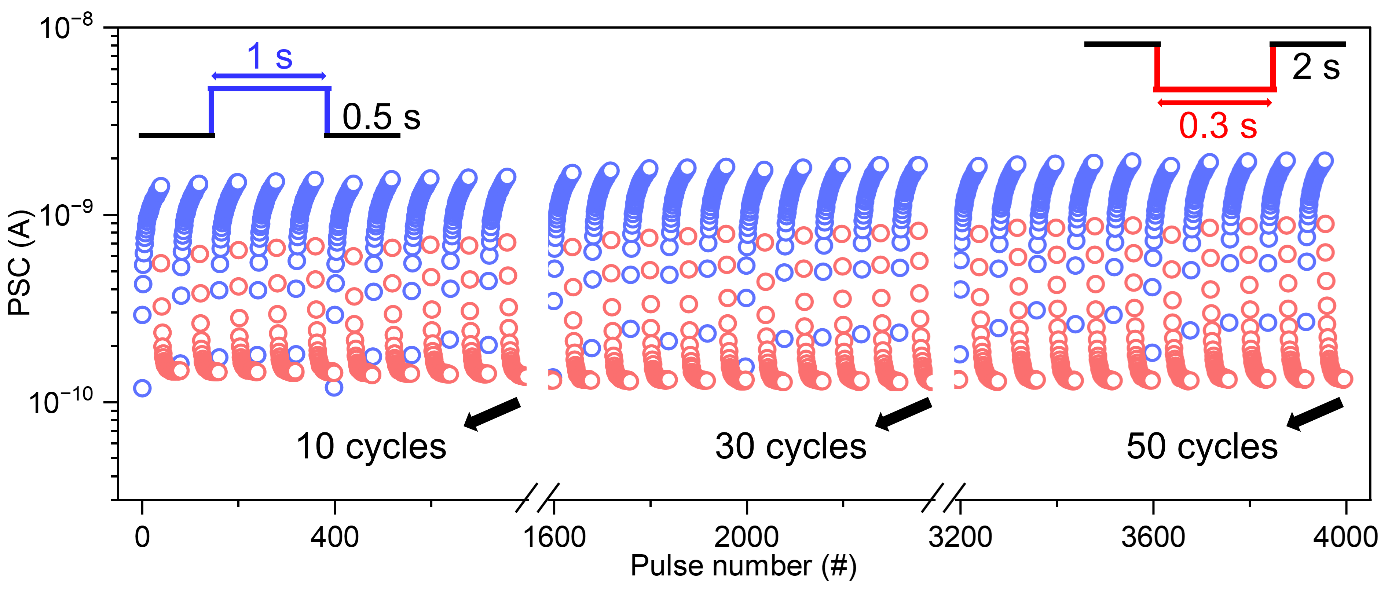


**Figure S14.** Potentiation and depression characteristics measured repeatedly for 50 cycles. *V*_ds_ and *V*_g_ were fixed at −5 V and 7 V. Potentiation and depression exhibit gradual modulation of PSC following each cycle.


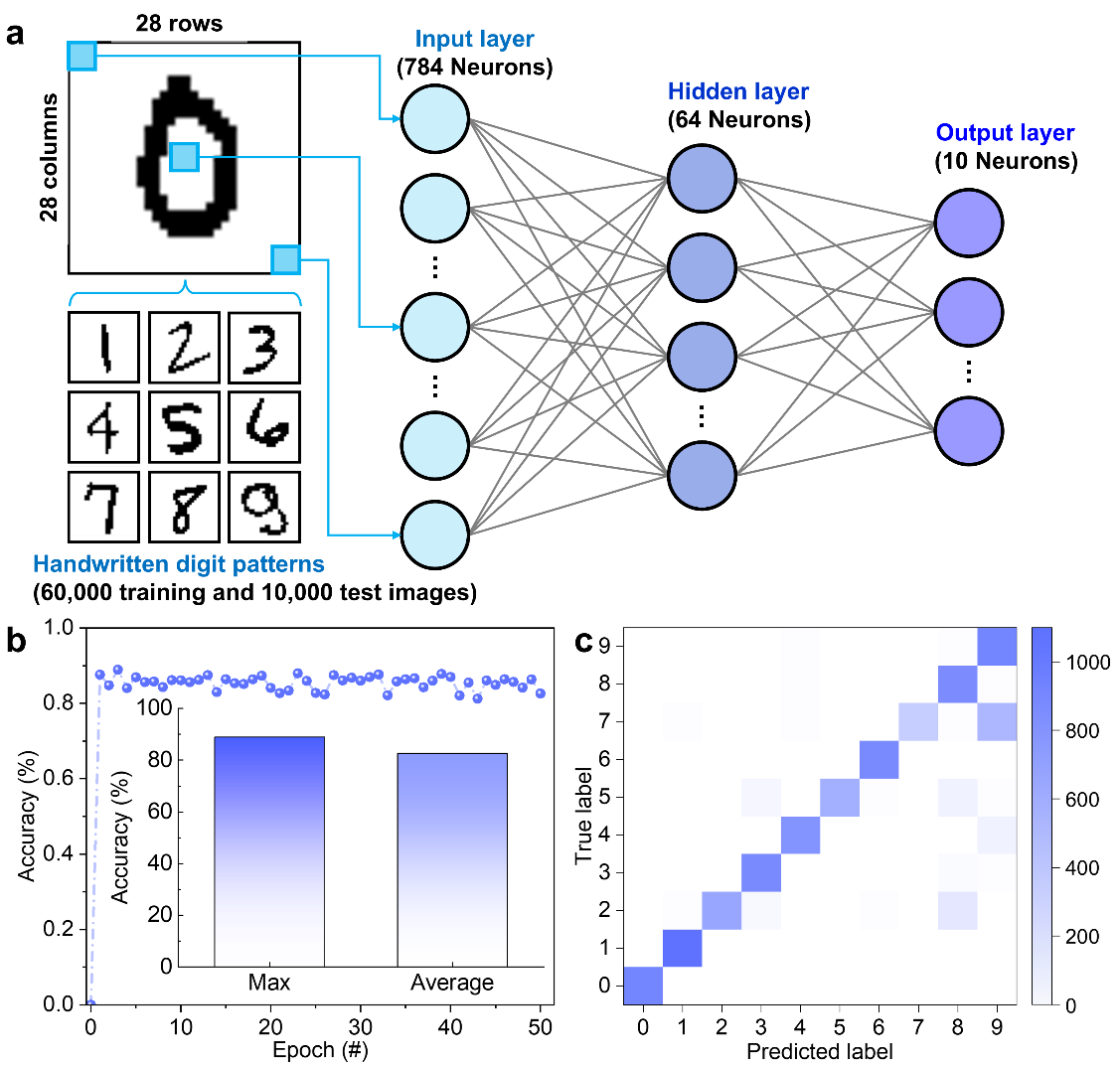


**Figure S15.** MNIST handwritten digit classification result; (a) Schematic of the ANN model with 784 input neurons (28 × 28 pixels), a single hidden layer of 64 neurons, and 10 output neurons corresponding to digit classes (0-9). A total of 60,000 training and 10,000 test images from the MNIST dataset were used to evaluate the classification performance of DPP-DTT NWs-based PT. (b) Training accuracy over 50 epochs for handwritten digit classification using the DPP-DTT NWs-based PT (inset: histogram showing maximum accuracy of 88.9% and average accuracy of 82.6%). (c) Confusion matrix for classification of 10 handwritten digits using 10,000 test images.


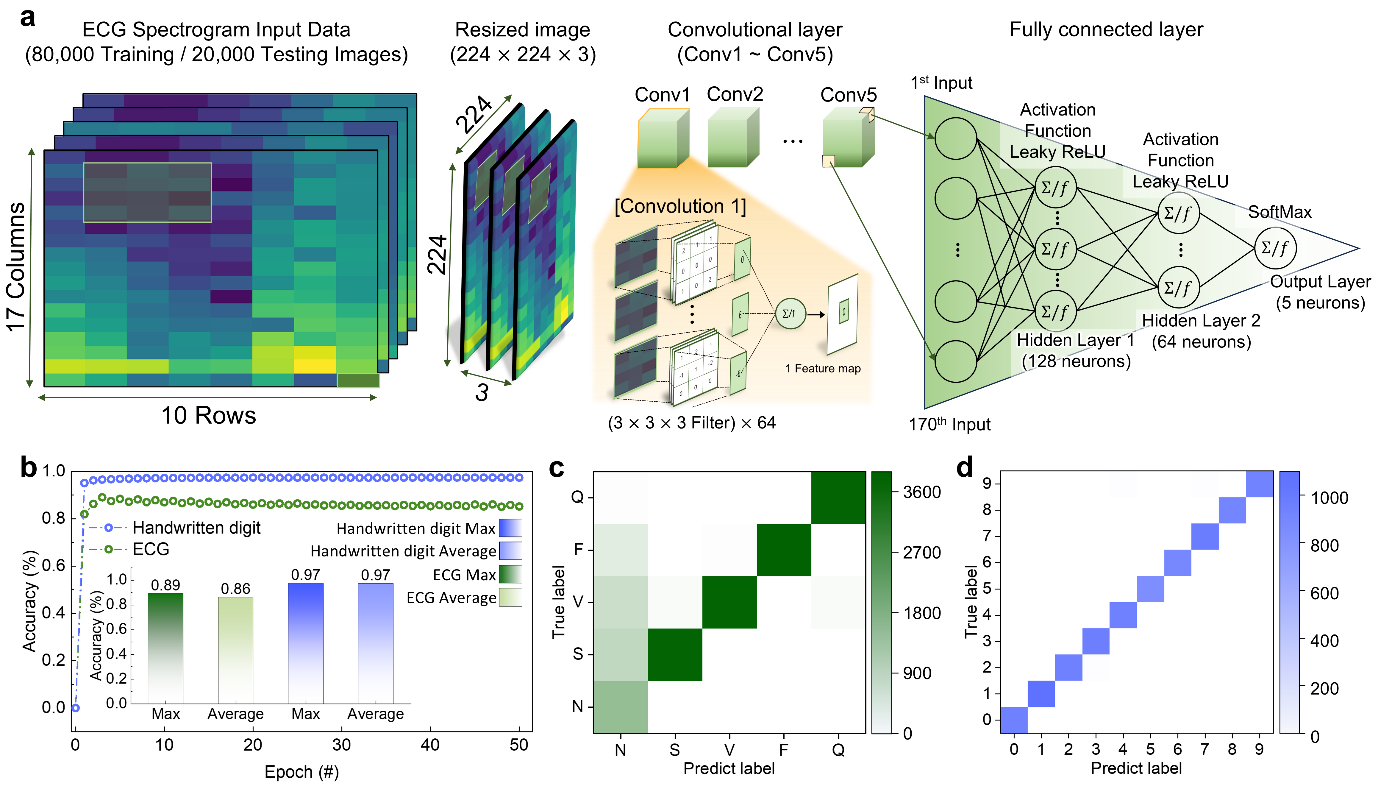


**Figure S16.** (a) Architecture of the VGG16 based CNN used for ECG spectrogram classification, consisting of five convolutional blocks and three fully connected layers with five classes. (b) Training accuracy using a CNN over 50 epochs for two datasets: ECG spectrogram (maximum accuracy: 89%, average accuracy: 86.2%) and MNIST handwritten digits (maximum accuracy: 97.4%, average accuracy: 97.2%). Confusion matrices for the classification test of (c) 5 ECG classes and (d) 10 handwritten digits.


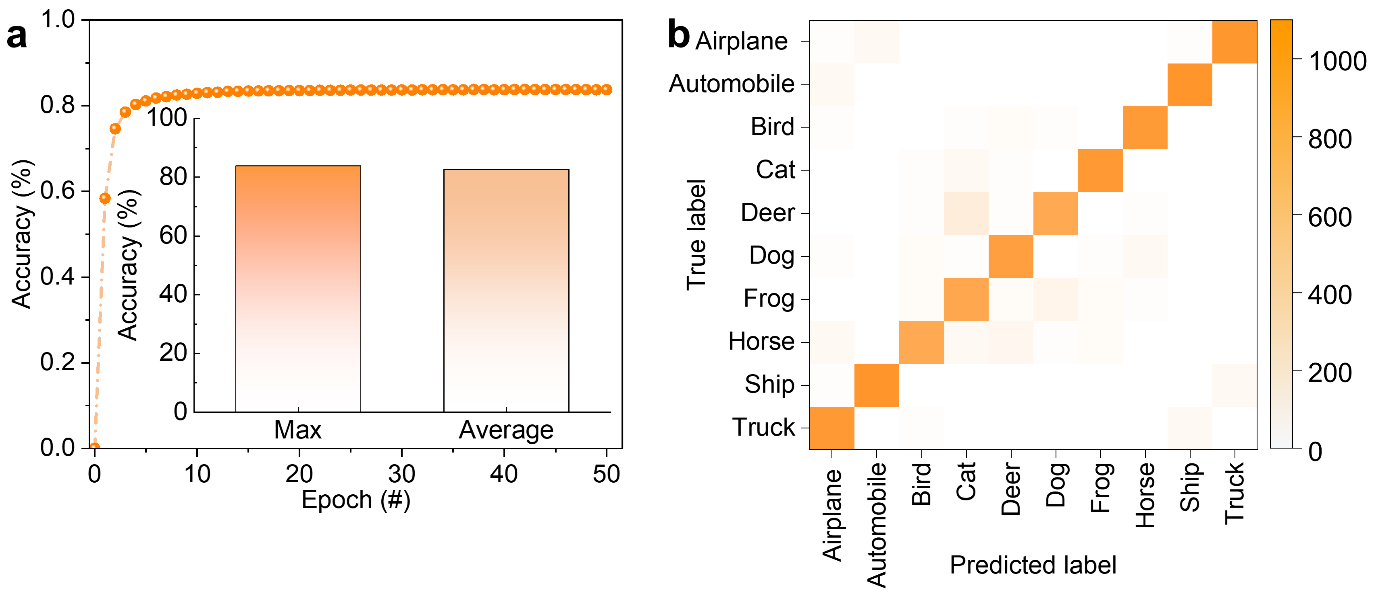


**Figure S17.** (a) Classification accuracy on the CIFAR-10 dataset using the CNN architecture trained for 50 epochs (maximum accuracy: 83.8%, average accuracy: 82.6%). (b) Confusion matrix for the classification test of CIFAR-10 dataset.

**
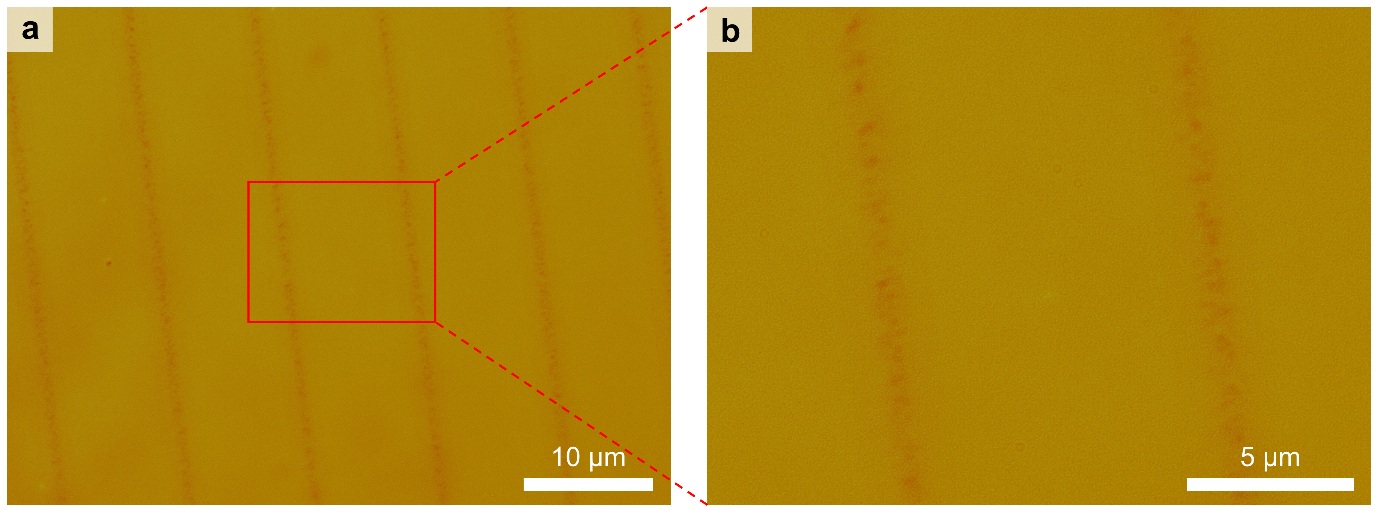
**

**Figure S18.** (a) OM image of DPP-DTT NWs formed on O_2_ plasma-treated SiO_2_ substate. (b) Enlarged OM image of the red-boxed region in Figure S18a, showing discontinuous one-dimensional dot-like features instead of continuous NWs due to the disruption of capillary force growth on the hydrophilic surface.

**Table S1**. Summary table of comparing trap-dependent parameters of DPP-DTT-based phototransistor with thin-film and NW geometries.

| **Device** | **Hysteresis**  **(V)** | **Subthreshold Swing**  **(V∙dec^−1^)** | ***D*_it_**  **(cm^−2^∙eV^−1^)** | **Photo-gated Shift**  **(V)** | **Photoresponsivity ^1^**  **(A∙W^−1^)** |
| --- | --- | --- | --- | --- | --- |
| DPP-DTT thin-film phototransistor | 5.15 | 1.41 | 4.89×10^12^ | 2.25 | 6.08×10^−6^ |
| DPP-DTT NW phototransistor | 5.95 | 1.54 | 5.38×10^12^ | 6.36 | 2.54×10^−2^ |

^1^ Photoresponsivity for the DPP-DTT NW and thin-film devices were extracted at *V*_g_ = 4 V and *V*_g_ = 3 V, respectively, corresponding to their maximum ratio.

| **Device**  **(Material)** | **Stimuli Source**  **(Potentiation)** | **Stimuli Source**  **(Depression)** | **# of Conductance States (LTP/LTD)** | **Cycle Durability** | **Network Model** | **Train Data** | **Recognition Accuracy (%)** | **Ref.** |
| --- | --- | --- | --- | --- | --- | --- | --- | --- |
| Memristor  (Zn_2_SnO_4_/Ga_2_O_3_) | Electrical | Electrical | 300/300 | 20 cycles | CNN | CIFAR-10 | 81.0 | [S1] |
| Memristor  (BDAPbI_4_)  (BDAZnI_4_) | Electrical  Electrical | Electrical  Electrical | 30/30  50/50 | N/A | CNN | CIFAR-10 | 83.0  81.0 | [S2] |
| c-NSFET ^1^  (Si, FG = Si_3_N_4_ ^2^)  e-NSFET ^3^  (Si, FG = Si_3_N_4_) | Electrical  Electrical | Electrical  Electrical | 32/32  32/32 | N/A | CNN | CIFAR-10 | 84.2  88.8 | [S3] |
| Phototransistor  (IDTBT@PC61BM) | Optical and Electrical  co-stimulation | Optical and Electrical  co-stimulation | 50/50 | N/A | SLP ^4^ | MNIST | 86 | [S4] |
| Phototransistor  (C8-BTBT@CsPbBr3@PS) | Optical | Electrical | 100/100 | N/A | SLP | MNIST | 75 | [S5] |
| Phototransistor  (DPP-DTT/PSPMA) | Optical | Electrical | 50/50 | N/A | MLP | MNIST | 93.3 | [S6] |
| Phototransistor  (DPP-DTT@Cl-HABI) | Optical | N/A | N/A | N/A | SLP  MLP | MNIST  MNIST | $\approx$ 80  85.5 | [S7] |
| Phototransistor  (DPP-DTT NWs) | Optical | Electrical | 40/40 | 50 cycles | MLP | MNIST  ECG  EMG | 88.9  76.8  93.4 | This work |
|  |  |  |  |  | CNN | MNIST  ECG  CIFAR-10 | 97.4  89  83.8 |  |

**Table S2.** Summary of synaptic devices with optical and electrical stimulus.

^1^ Conventional vertically stacked nanosheet field effect transistor, ^2^ Floating-gate = Si_3_N_4_, ^3^ Engineered vertically stacked nanosheet field effect transistor, ^4^ Single layer perceptron.

| **Device**  **(Material)** | **Wavelength**  **(Range)** | **Stimuli Source**  **(Potentiation)** | **Stimuli Source**  **(Depression)** | **# of Conductance States (LTP/LTD)** | **Cycle Durability** | **Network Model** | **Train Data** | **Recognition Accuracy (%)** | **Ref.** |
| --- | --- | --- | --- | --- | --- | --- | --- | --- | --- |
| Phototransistor  (DPP-DTT/PSPMA) | 365 nm  (UV ^1^) | Optical | Electrical | 50/50 | N/A | MLP | MNIST | 93.3 | [R6] |
| Phototransistor  (DPP-DTT/PCBM@PAN) | 365 nm  (UV) | Optical | Electrical | 25/25 | N/A | MLP | MNIST | 95 | [R8] |
| Phototransistor  (DPP-DTT@Cl-HABI) | 240–400 nm (UV) | Optical | N/A | N/A | N/A | SLP ^2^  MLP | MNIST  MNIST | $\approx$ 80  85.5 | [R7] |
| Photomemory  (DPP-DTT, FG = AlO_x_ NP ^3^) | 365–620 nm  (UV-Vis ^4^) | Optical | Electrical | 40/40 | 3 cycles | MLP | MNIST | 93.2 | [R9] |
| Phototransistor  (DPP-DTT NWs) | 455–660 nm  (Vis) | Optical | Electrical | 40/40 | 50 cycles | MLP | MNIST  ECG  EMG | 88.9  76.8  93.4 | This work |
|  |  |  |  |  |  | CNN | MNIST  ECG  CIFAR-10 | 97.4  89  83.8 |  |

**Table S3**. Summary of synaptic performance for DPP-DTT-based devices under various structural and optical conditions.

^1^ Ultraviolet, ^2^ Single layer perceptron, ^3^ Floating-gate = AlO_x_ nanoparticle, ^4^ Ultraviolet-visible.

**Supporting Reference**

[S1] Shrivastava, S.; Dai, W. S.; Limantoro, S. E.; Juliano, H.; Tseng, T. Y., *Advanced Electronic Materials* **2025,** *11* (3), 2400527.

[S2] Patel, M.; Gosai, J.; Khemnani, M.; Andola, B.; Srivastava, Y. K.; Lamba, T. K.; Ranjan, M.; Solanki, A., *Appl. Mater. Today.* **2025,** *44*, 102784.

[S3] Raza Ansari et al. *ACS Applied Electronic Materials* 2023, 5, 12, 7079-7086.

[S4] Raza Ansari, M. H.; Navlakha, N.; El-Atab, N., *ACS Applied Electronic Materials* **2023,** *5* (12), 7079-7086.

[S5] Shi, Q.; Liu, D.; Hao, D.; Zhang, J.; Tian, L.; Xiong, L.; Huang, J., *Nano Energy* **2021,** *87*, 106197.

[S6] Wu, J.; Wang, X.; Tang, X.; Liang, Z.; Hu, B.; Ran, Y.; Bu, L.; Lu, G., *Adv. Funct. Mater.* **2025**, 2420073.

[S7] Liu, D.; Zhang, J.; Shi, Q.; Sun, T.; Xu, Y.; Li, L.; Tian, L.; Xiong, L.; Zhang, J.; Huang, J., *Adv. Mater.* **2024,** *36* (1), 2305370.

[S8] Liang, Z.; Wang, X.; Song, Z.; Wang, Q.; Tang, X.; Wu, J.; Bu, L.; Lu, G., *ACS Appl. Mater. Interfaces* **2024,** *16* (47), 65091-65099.

[S9] Wang, X.; Yang, S.; Qin, Z.; Hu, B.; Bu, L.; Lu, G., *Adv. Mater.* **2023,** *35* (40), 2303699.
